# How is nicotine vaping product (e-cigarette) use monitored in primary care electronic health records in the United Kingdom? An exploratory analysis of Clinical Practice Research Datalink (CPRD)

**DOI:** 10.1186/s12889-023-17200-7

**Published:** 2023-11-16

**Authors:** Bernadett E. Tildy, Ann McNeill, John Robins, Alexandru Dregan, Sol Richardson, Leonie S. Brose

**Affiliations:** 1 Addictions Department, Institute of Psychiatry, Psychology & Neuroscience (IoPPN), King’s College London, Addiction Sciences Building, 4 Windsor Walk, London, SE5 8BB UK; 2 SPECTRUM Consortium, London, UK; 3 NIHR Applied Research Collaboration South London, London, UK; 4 Psychological Medicine Department, Institute of Psychiatry, Psychology & Neuroscience (IoPPN), King’s College London, 16 De Crespigny Park, London, SE5 8AF UK; 5 Vanke School of Public Health, Tsinghua University, Mingli Building, Haidian District, Beijing, 100083 China

**Keywords:** Smoking, Tobacco, Smoking cessation, Substance use, Vaping product, Nicotine, e-cigarette, General practice, Family practice, Family medicine, Primary care, Electronic health records, CPRD

## Abstract

**Background:**

Electronic health records (EHRs) could identify long-term health effects of nicotine vaping. We characterised the extent to which vaping is recorded in primary care EHRs in the UK, on a population level.

**Methods:**

We performed descriptive analysis of Clinical Practice Research Datalink (CPRD), primary care electronic health records of 25% of the UK population (~ 16 million patients). Patients aged ≥ 18 years whose vaping status was recorded using medical codes between 2006 and 2022 were identified. We reported the frequency of vaping codes; their distribution by patient age, gender, and ethnicity; trends in vaping recording over time (including interrupted time series analyses); and transitions in patient smoking status.

**Results:**

Seven medical codes indicated current or former vaping, from 150,114 patients. When their vaping status was first recorded, mean patient age was 50.2 years (standard deviation: 15.0), 52.4% were female, and 82.1% were White. Of those recorded as currently vaping, almost all (98.9%) had records of their prior smoking status: 55.0% had been smoking, 38.3% had stopped smoking, 5.6% had never smoked. Of those who were smoking prior to being recorded as vaping, more than a year after the vaping record, over a third (34.2%) were still smoking, under a quarter (23.7%) quit smoking, 1.7% received a ‘never smoked’ status, and there was no smoking status for 40.4%. The ‘e-cigarette or vaping product use-associated lung injury’ (EVALI) outbreak was significantly associated with a declining trend in new records of current vaping between September 2019 and March 2020; and an immediate significant increase in new records of former vaping, followed by a declining trend.

**Conclusions:**

Few patients are being asked about vaping. Most who vape had smoked, and many quit smoking after starting vaping. To enable electronic health records to provide stronger evidence on health effects, we recommend improved completeness, accuracy and consistency.

**Supplementary Information:**

The online version contains supplementary material available at 10.1186/s12889-023-17200-7.

## Background

Smoking is a leading preventable cause of illness and premature death in the United Kingdom (UK) and worldwide [1]. Evidence suggests that using nicotine vaping products (NVPs, or e-cigarettes) is less harmful than smoking tobacco [2], and NVPs improve smoking cessation likelihood compared to nicotine replacement therapy [3]. However, due to uncertainty about the long-term health effects of NVPs and concerns around youth uptake, policy and guidelines around NVPs vary internationally [4]. Some clinical guidelines recommend that health professionals encourage the use of NVPs as another option for smoking cessation on a par with medicinally licensed pharmacotherapies and behavioural support [4, 5]. For example, the UK National Institute for Health and Care Excellence clinical guidelines recommend that adults who smoke have access to NVPs alongside other smoking cessation interventions [6]. In the UK, NVPs are regulated as consumer products and NVPs are available on the open market to those aged ≥ 18 years [2]. NVPs are the most popular smoking cessation aid in the UK [2] and 9.1% of adults in Great Britain regularly used NVPs in 2023 [7].

Monitoring NVP use prevalence and uptake can establish the long-term benefits and harms of NVP use [5]. Although population surveys can generate NVP use prevalence estimates [7–9], these are often cross-sectional, under-sample vulnerable populations, have short-term follow-up, or do not enquire extensively about health outcomes. Electronic health records (EHRs) could help identify long-term health effects of NVP use, pending NVP use data completeness.

Currently, studies about how health professionals are documenting NVP use in EHRs are limited (Supplementary Box 1). Studies from the United States (US), found low vaping screening rates in EHRs, ranging from: 14.3% in 2019 [10] to 34.8% in 2021–2022 [11]; 0% in 11–17-year-olds in 2016–2017 [12] and 16% in 18–35-year-olds who had never smoked in 2020 [13]. US studies found that patients with documented (current) vaping were more likely to be male, aged 18–44 years, and White [14, 15]. Although the prevalence of US patients who have vaping documentation is still low (< 1%), it is increasing. First-time incidence of vaping documentation increased from 0.1 to 95 per 100,000 patients from 2006 to 2015 [14, 16] and the prevalence of vaping documentation (including ‘never vaping’) increased from 0.0032 to 0.46% in progress notes between 2009 and 2014 [17]. Similar to population surveys, the rate of current/former vaping in non-smoking populations is relatively low in EHRs [13]; patients are more likely to be screened for vaping if they have indicated that they smoke [11], hence there are high proportions of current and former smoking among those who have vaping documentation [14, 16, 17]. Two US studies used EHRs (2012–2015 [16], 2018–2020 [15]) to examine transitions between current vaping and smoking status, finding that smoking cessation was more likely among those who received current vaping documentation compared to those not vaping.

To our knowledge, there have been no studies specifically investigating health professionals’ documentation of vaping in the UK. In the UK, general practitioners (GPs) are required to record standardised information on clinical conditions, such as smoking status. Although a 2018 Royal College of Physicians (UK) consultation recommended NVP use recording in EHRs [18], GPs are not currently incentivised to record this via the pay-for-performance scheme (Quality and Outcomes Framework, QOF). UK QOF guidelines (2018/19–2022/23 [19]) recommend that NVP users “who have never smoked or given up smoking should be classified as non-smokers or ex-smokers respectively”, which may lead to under-recording of NVP use in EHRs. Other UK guidance (2020 [20], 2021 [6]) recommended that health professionals ask about NVP use routinely. This guidance was in response to an outbreak of severe lung injuries largely confined to the US: ‘e-cigarette or vaping product use-associated lung injury’ (EVALI) [21]. EVALI was purported to be associated with conventional nicotine vaping, but the US CDC concluded that vaping products which contained tetrahydrocannabinol and Vitamin E acetate were linked to most cases [2, 21]. EVALI was identified in July 2019, followed by a peak in cases and US news coverage [22] in September 2019, then a steady decline through early 2020 [21].

It is not known to what extent vaping is recorded in UK EHRs. The use of existing medical codes to record vaping is hypothesised to be suboptimal [23]. We aimed to describe and characterise the extent to which NVP use is being recorded in primary care in the UK, using Clinical Practice Research Datalink (CPRD) data.

### Research questions (RQs)

RQ1. Which medical codes indicative of current vaping and former vaping are most frequently used in primary care EHRs in the UK between 2006 and March 2022?

RQ2. What are temporal trends in the first-time incidence of current and former vaping codes, and was there a change in the incidence pre- and post-EVALI outbreak in the US?

RQ3. How does the distribution of vaping codes vary with patient demographics (age, gender, ethnicity)?

RQ4. What are the transitions in smoking status among patients who received their first current or former vaping code, comparing previous and subsequent (> 12 months) smoking status records?

## Methods

### Data source

CPRD includes anonymised medical records from UK general practices from 1990 to the present [24]. CPRD includes detailed medical data for approximately 16 million active patients (25% of the UK population) and 60 million historical patients from around 2,000 UK practices (26% of UK practices). The dataset is representative of the UK population in terms of geography, relative social deprivation, age and gender [25]. In a recent CPRD dataset (linked with Hospital Episode Statistics), over 80% of currently registered patients had their ethnicity recorded and the distribution was broadly representative of the UK population [26]. Prevalence estimates from 2007–2011 CPRD data for current smoking, and non-smoking, were found to be similar to those from nationally representative surveys [27]. CPRD collects diagnostic, therapeutic, laboratory, referral, and demographic data from GP practices on a monthly basis [24]. For this study, we pooled data from the CPRD GOLD April 2023 build and the CPRD Aurum March 2023 build; both had a cut-off event date of 31 March 2022, because CPRD was experiencing temporary issues with data quality after this date [28].

### Recording vaping product use

GPs can record a vaping event in EHRs via specific SNOMED or Read medical codes during consultations with patients [24], these codes are not carried forward automatically to future consultation records. GPs can also save free-text comments, but these are not available for research purposes.

### Patient population

All patients (aged ≥ 18 years at the date of the consultation) who received a code related to vaping at any point (‘incidence’) from 1 September 2006 to 31 March 2022 were extracted. Records from patients classified as ‘acceptable’ by CPRD were included (those with a valid gender and birth date; and logically consistent and valid registration and transferred-out dates). Records from ‘up-to-standard practices’ (CPRD GOLD quality marker) were included. Duplicate records were excluded, following data specification documents [28].

After exclusions (Supplementary Fig. 1), the analytical sample included 225,111 observations from 150,114 unique patients. In total, there were 152,277 first-time incident events of current or former vaping codes: 147,130 patients ever received a current vaping code, 5,147 patients ever received a former vaping code, and 2,163 received both codes (Supplementary Fig. 2).

### Outcome

Our main outcome variable was the incidence of codes indicating current or former vaping. Using the CPRD medical code browser, we identified ten codes used between 1 September 2006 and 31 March 2022 which relate to electronic cigarettes/e-cigarettes, vaping/vaper, electronic nicotine delivery systems (ENDS), and e-liquid (Supplementary Table 1). We derived a new variable which aggregated seven of the codes which indicated ‘current vaping’ or ‘former vaping’ specifically (Table 1).

**Table 1:** Frequency of current vaping and former vaping codes

Derived vaping code variable	Medical code term in CPRD	Dataset	Freq (n)	Month, year first used	Freq (n)
**Current vaping**	Electronic cigarette user	Aurum	212,522	Oct 2011	219,478
	User of electronic cigarette	GOLD	6,940	Oct 2013	
	User of electronic cigarette	Aurum	13	Oct 2013	
	e-cigarette user	Aurum	3	Dec 2019	
	Vaper with nicotine	Aurum	0	NA	
**Former vaping**	Ex user of electronic cigarette	Aurum	5,587	Feb 2014	5,633
	Ex user of electronic cigarette	GOLD	46	Sept 2015	
**TOTAL observations**					225,111

We identified 10 medical codes used between 1 September 2006 and 31 March 2022 (Supplementary Table 1). We derived a new variable which aggregated seven of the codes which indicated ‘current vaping’ or ‘former vaping’. Three ambiguous medical codes (“e-cigarette”, “Electronic cigarette”, “Electronic cigarette liquid”, n = 57 observations) were excluded

### Covariates

Covariates included: patient gender (male, female, non-binary/unknown), patient age when they received the vaping code (year of birth minus event date of consultation where the patient received the vaping code), geographical region of the patient’s practice (North East, North West, Yorkshire and The Humber, East Midlands, West Midlands, East of England, London, South East, South West, Wales, Scotland, Northern Ireland), patient ethnicity (Asian, Black, Mixed, White, Other, unknown) and patient smoking status (never smoked, currently smoke, formerly smoked, unknown).

Ethnicity was coded using higher-level UK Census 2011 Ethnicity Categories [26]. We mapped ethnicity and smoking status-related codes to classifications used in previous studies (Supplementary Tables 2a, 2b, 3a, 3b). Ethnicity and smoking status data recorded prior to when a patient was 18 years old were retained.

For each first-time incidence of a vaping code, we sought to derive a current smoking status at three time points:

1. **Previous smoking status**: smoking status that was recorded in the patient consultation that chronologically immediately preceded receiving a vaping code.
2. **Concurrent smoking status**: smoking status that was recorded in the patient consultation that occurred on the same date as receiving a vaping code.
3. **Subsequent smoking status**: smoking status that was recorded in the chronologically latest patient consultation that took place > 12 months (> 365 days) after receiving a vaping code, to capture a long-term smoking cessation outcome.

Where a patient had multiple records of smoking status in their preceding, concurrent or subsequent consultation, if any of the smoking status records were ‘currently smoking’, this was designated as the smoking status for the respective time period.

### Data analysis

Analyses were conducted in R (version 4.2.1), except the interrupted time-series analysis which was conducted in Stata 17.

RQ1: We used descriptive statistics to report the frequency of vaping codes classified as current vaping or former vaping. We calculated the number of unique patients receiving one or more vaping codes over time.

RQ2: To characterise trends in patient-level first-time incidence of vaping codes, if a unique patient had multiple consultations over time where they received a current vaping or former vaping code, only the first instance (earliest) of a particular vaping code (i.e., current vaping or former vaping) was included in the frequency count for that particular code (similar to previous work [14, 16]). Following this, using CPRD denominator files (Supplementary Box 2), patient-level proportions of vaping code first-time incidence over time were calculated by dividing the number of current/former vaping patients, by the denominator (all eligible patients contributing data to CPRD), per month. We also calculated patient-level proportions of vaping code first-time incidence over time, by geographical region.

To investigate any pre- and post-EVALI outbreak effects, we performed single-group ordinary least-squares interrupted time-series analysis using the Stata package *itsa* [29]. We fitted two models with monthly numbers of current and former vaping status records from August 2015 (when a government-commissioned report [30] on vaping increased discussion around vaping) to January 2022 as their dependent variables. Time was fitted as a linear variable representing months since September 2011. Two interruptions were modelled as occurring in September 2019 (month 97), corresponding to the peak of US media coverage about EVALI [22]; and April 2020 (month 104), corresponding to the start of the first national Coronavirus disease (COVID-19) pandemic lockdown in the UK. Models were fitted with a maximum lag term of 12 months to account for autocorrelation in the dependent variable. Both models were also adjusted for monthly numbers of all eligible patients contributing data to CPRD as a linear variable. Results were expressed as regression coefficients for the change in monthly numbers of new current or former vaping records, with Newey–West standard errors accounting for autocorrelation and potential heteroskedasticity.

RQ3: Using our patient-level first-time incidence of vaping codes, we used descriptive statistics to report the proportions of patients who received a vaping code by age, gender, and ethnicity.

RQ4: Using our patient-level first-time incidence of vaping codes, we reported the concurrent smoking status for patients who received a current vaping or former vaping code. We plotted previous smoking status and subsequent (> 12 month) smoking status, separately for first-time current and former vaping, to describe transitions in smoking status over time. As a sensitivity analysis, we plotted a supplementary graph where the subsequent smoking status was the smoking status recorded in the chronologically latest patient consultation that took place > 12 months (> 365 days) but ≤ 24 months (≤ 730 days) after receiving the first-time current vaping code.

### Ethical approval

The study protocol was granted scientific and ethical approval by the Medicine and Healthcare Regulatory Agency Independent Scientific Advisory Committee (ISAC: Protocol No. 21_000706).

## Results

### RQ1: Medical codes indicating current vaping and former vaping

Of the seven codes indicating current vaping or former vaping, the “Electronic cigarette user” code in the CPRD Aurum dataset was the first (13 October 2011) and most frequently used; the “Vaper with nicotine” code was not used at all (Table 1). There were 219,478 consultations where a patient received a current vaping code and 5,633 consultations where a patient received a former vaping code.

Of 150,114 unique patients, 107,901 (71.9%) received only one code; 42,213 (28.1%) received multiple vaping codes, including 1,857 (1.2%) receiving more than five (Supplementary Table 4). There were 2,163 (1.4%) unique patients who had ever received both a current vaping and former vaping code – of these, 1,677 patients received a current vaping code before they received a former vaping code, and 486 vice versa. For those who received both a current and former vaping code, the mean time difference between receiving their first vaping code and their second was 729.0 days (standard deviation [SD]: 558.4), median: 616.0, range: 0.0–2,710.0.

### RQ2: Temporal trends and EVALI outbreak

#### Temporal trends

Across the 150,114 unique patients, there were 152,277 first-time incident events of current or former vaping codes: 147,130 patients ever received a current vaping code, 5,147 patients ever received a former vaping code, and 2,163 received both codes (Supplementary Fig. 2).

**Fig. 1:**
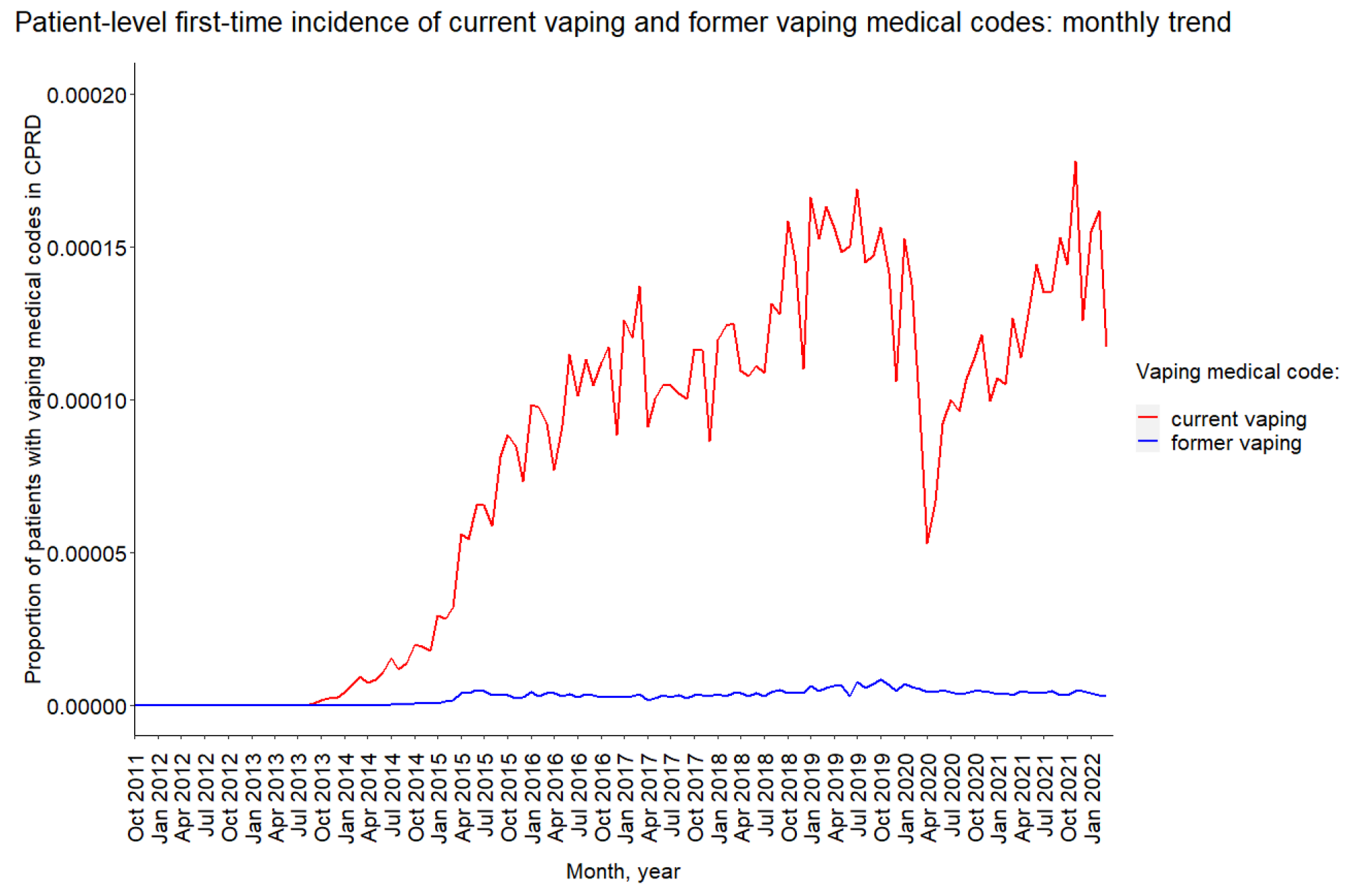
Patient-level first-time incidence of current vaping and former vaping medical codes Graph showing the proportion of patients who received a vaping code indicating first-time current vaping or former vaping out of all patients (≥ 18 years old) in CPRD that month, per month

Figure 1 shows the proportion of patients who received a vaping code indicating first-time current vaping or former vaping out of all patients (≥ 18 years old) in CPRD that month, per month. (Supplementary Graph 1 shows the trend by geographical region.) First-time incidence of vaping codes increased from September 2013. There was apparent seasonality, with a decrease in incidence during April and December. There was a notable decrease in incidence of current and former vaping codes in April 2020, the first month fully affected by the first Coronavirus disease (COVID-19) pandemic lockdown in the UK when of GP consultation frequency reduced significantly. Peak first-time incidence of current vaping codes was in November 2021: 17.8 per 100,000 patients contributing data to CPRD. Peak first-time incidence of former vaping codes was in October 2019: 0.9 per 100,0000 patients.

#### Interrupted time series analysis

The Breusch-Godfrey test for autocorrelation indicated that autocorrelation was present at up to eight months of lag for current vaping record outcomes and 12 months for former vaping record outcomes; these results suggested that the models appropriately accounted for autocorrelation.

**Fig. 2:**
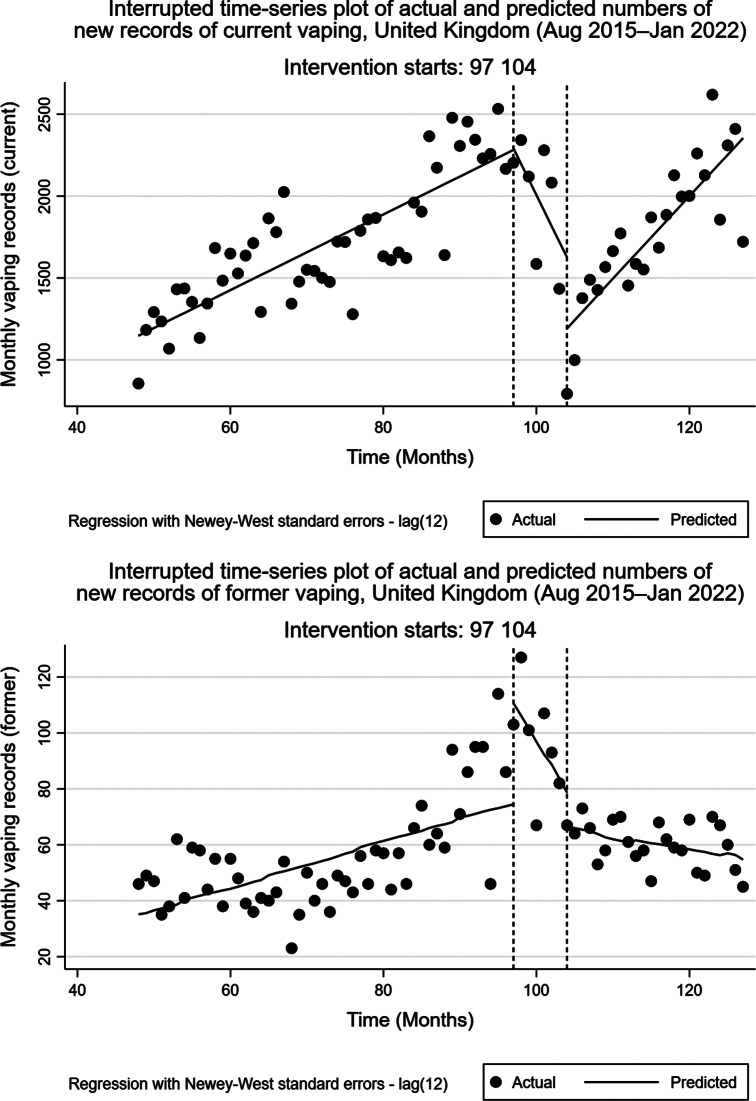
Interrupted time-series plot of current and former vaping records (August 2015 to January 2022). (**a**) Current vaping records. (**b**) Former vaping records

Figure 2a shows the interrupted time-series postestimation plot for current vaping record outcomes, including actual and predicted numbers of new monthly records. Model outputs showed that numbers of new current vaping records increased at a rate of 23.2 (95% CI: 14.1–32.2, p < 0.001) per month over the period analysed. After the peak of media coverage on EVALI, there was no significant step change in monthly numbers of new current vaping records (regression coefficient: 10.5, 95% CI: -221.1–242.1, p = 0.928). However, we found a significant change in the linear time trend in monthly numbers of new current vaping records, with a post-interruption linear time trend of -95.1 per month (95% CI: -122.6–-67.6, p < 0.001), between September 2019 and March 2020. After implementation of the first COVID-19 lockdown in the UK, there was a post-interruption decrease in monthly new current vaping records of -434.4 (95% CI: -738.9–-130.0, p = 0.006) followed by a rising post-interruption time trend in monthly numbers of records of 50.4 (95% CI: 38.9–62.0, p < 0.001) per month.

Figure 2b shows the interrupted time-series postestimation plot for new former vaping record outcomes, including actual and predicted numbers of monthly records. Model outputs showed that numbers of new former vaping records increased at a rate of 0.76 (95% CI: 0.10–1.43, p = 0.025) per month over the period analysed. After the peak of media coverage on EVALI, there was a statistically-significant step change in numbers of monthly numbers of new former vaping records (regression coefficient: 36.2, 95% CI: 17.9–54.4, p < 0.001). We found a significant change in the linear time trend in monthly numbers of new former vaping records, with a post-interruption linear time trend of -4.6 per month (95% CI: -5.8–-3.3, p < 0.001), between September 2019 and March 2020. After implementation of the first COVID-19 lockdown in the UK, there was a post-interruption decrease in monthly new former vaping records of -12.2 (95% CI: -20.3–-4.1, p < 0.001) followed by a gradually declining post-interruption time trend in monthly numbers of records of -0.4 (95% CI: -0.7–-0.2, p < 0.001) per month.

### RQ3: Distribution of vaping codes by patient demographics: age, gender, ethnicity

**Fig. 3:**
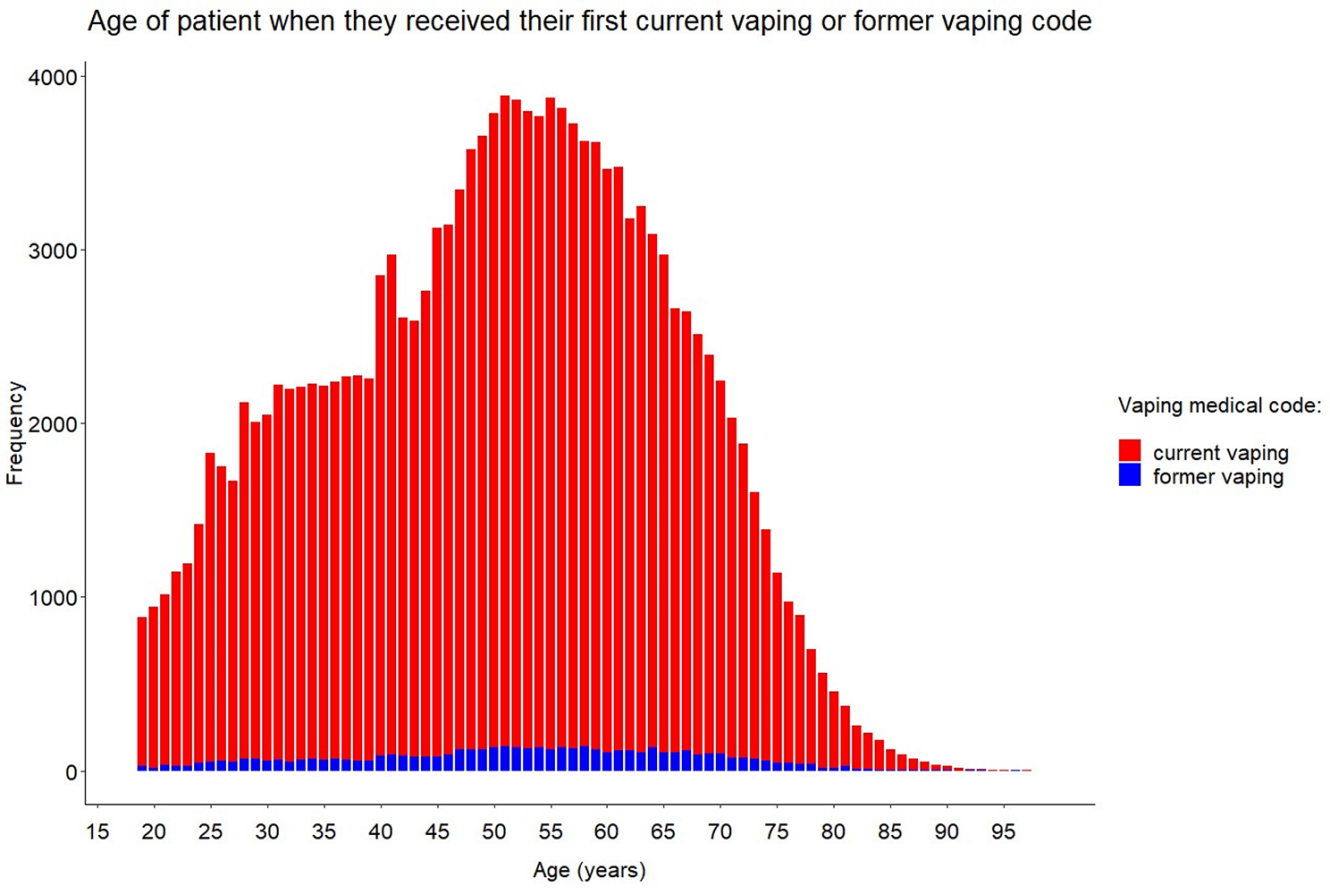
Age of patient when they received their first current vaping or former vaping code Graph showing the frequency of patients who received a vaping code indicating first-time current vaping or former vaping in CPRD, by the patient age at the time of receipt of the vaping code

The mean age of patients when they received their first current vaping code was 50.2 years (SD: 15.0, median: 51.0, range: 18.0–99.0), and 52.2 years (SD: 15.0, median: 53.0, range: 18.0–96.0) when they received their first former vaping code (Fig. 3).

The gender distribution in our sample was approximately balanced: 52.4% female, 47.7% male (Table 2).

**Table 2:** Gender and ethnicity of patients who received a vaping medical code

Patient characteristic	Current vaping code, n (%) 147,130 (100.0)	Former vaping code, n (%) 5,147 (100.0)	Current or Former vaping code, n (%) 150,114 (100.0)
**Gender**			
Male	69,993 (47.6)	2,615 (50.8)	71,538 (47.7)
Female	77,133 (52.4)	2,532 (49.2)	78,572 (52.3)
Indeterminate	4 (0.0)	0 (0.0)	4 (0.0)
**Ethnicity**			
Asian	3,367 (2.3)	121 (2.4)	3,449 (2.3)
Black	1,320 (0.9)	47 (0.9)	1,358 (0.9)
Mixed	781 (0.5)	28 (0.5)	803 (0.5)
Other	2,319 (1.6)	44 (0.9)	2,344 (1.6)
White	120,790 (82.1)	4,426 (86.0)	123,310 (82.1)
Unknown	18,553 (12.6)	481 (9.4)	18,850 (12.6)

Table showing the frequency and proportion of patients who received a vaping code between 1 September 2006 and 31 March 2022 by gender and ethnicity

Of 150,114 unique patients, ethnicity was recorded as ‘unknown’ for 18,553 (12.6%). The high-level ethnicity categories of patients who received a vaping code were: 2.3% Asian, 0.9% Black, 0.5% Mixed, 1.6% Other, 82.1% White and 12.6% unknown (Table 2).

### RQ4: Smoking status transitions among patients who received a vaping code

Of 150,114 unique patients, 149,624 had at least one smoking status record, while 490 (0.3%) had no smoking status record (unknown).

#### Concurrent Smoking status

**Fig. 4:**
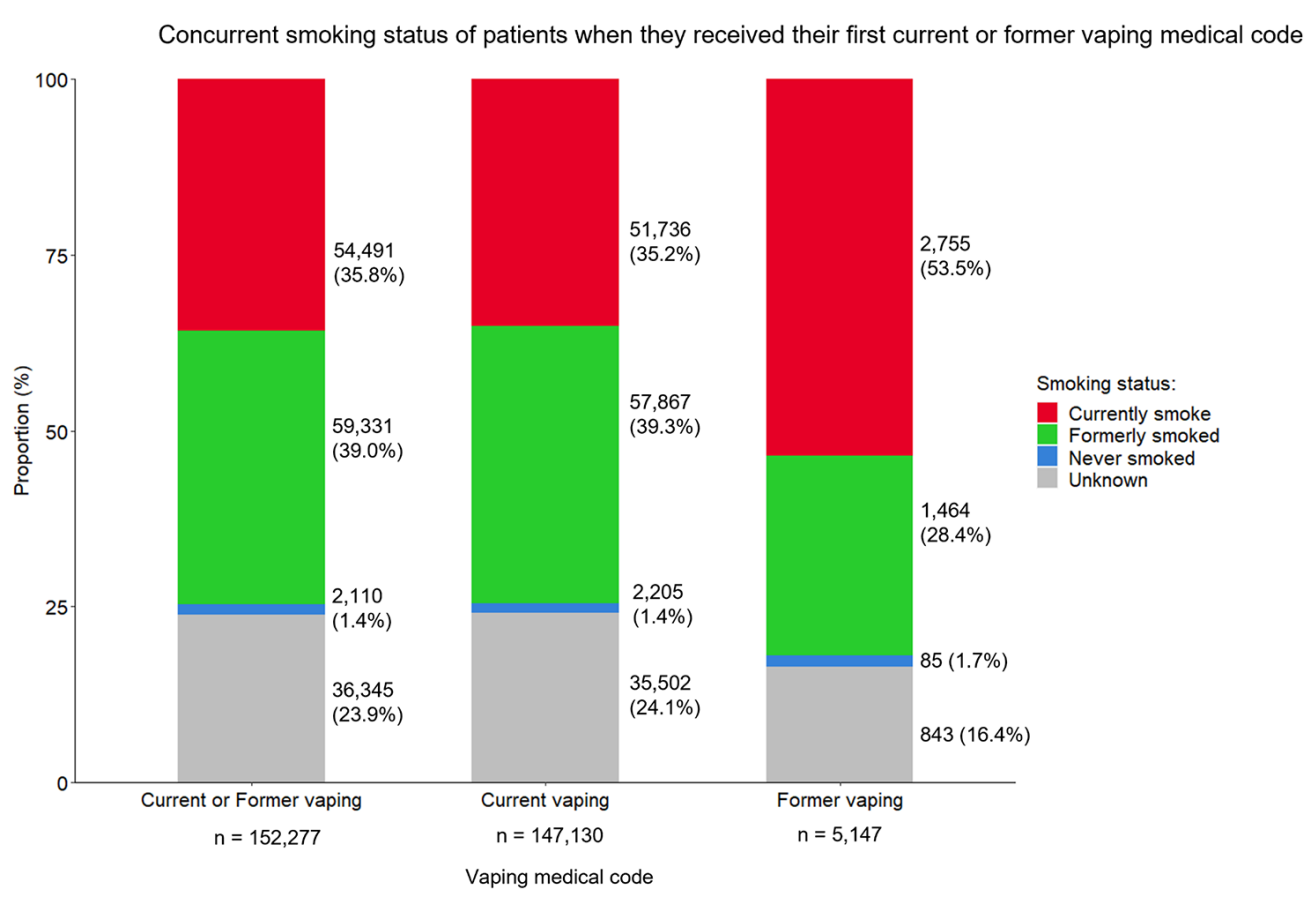
Concurrent smoking status of patients when they received their first current or former vaping code Graph showing the concurrent smoking status of patients when they received a vaping code indicating first-time current vaping or former vaping. Concurrent smoking status: the smoking status that was recorded for the patient on the same date as when the patient received the first-time current or former vaping code

Over three quarters (115,932/152,277, 76.1%) of patients had their concurrent smoking status recorded within the same consultation when they first received any (current/former) vaping code (Fig. 4). Of these, the majority (n = 113,822, 98.2%) were either currently smoking (n = 54,491, 47.0%) or had quit smoking in the past (n = 59,331, 51.2%), and those recorded as having never smoked comprised a small proportion (n = 2,110, 1.8%). These proportions were similar for those who received a current vaping code. Among those who received a former vaping code, a larger proportion were currently smoking compared to among those who received a current vaping code (53.5% vs. 35.2%).

#### Smoking status transitions

##### Current vaping

**Fig. 5:**
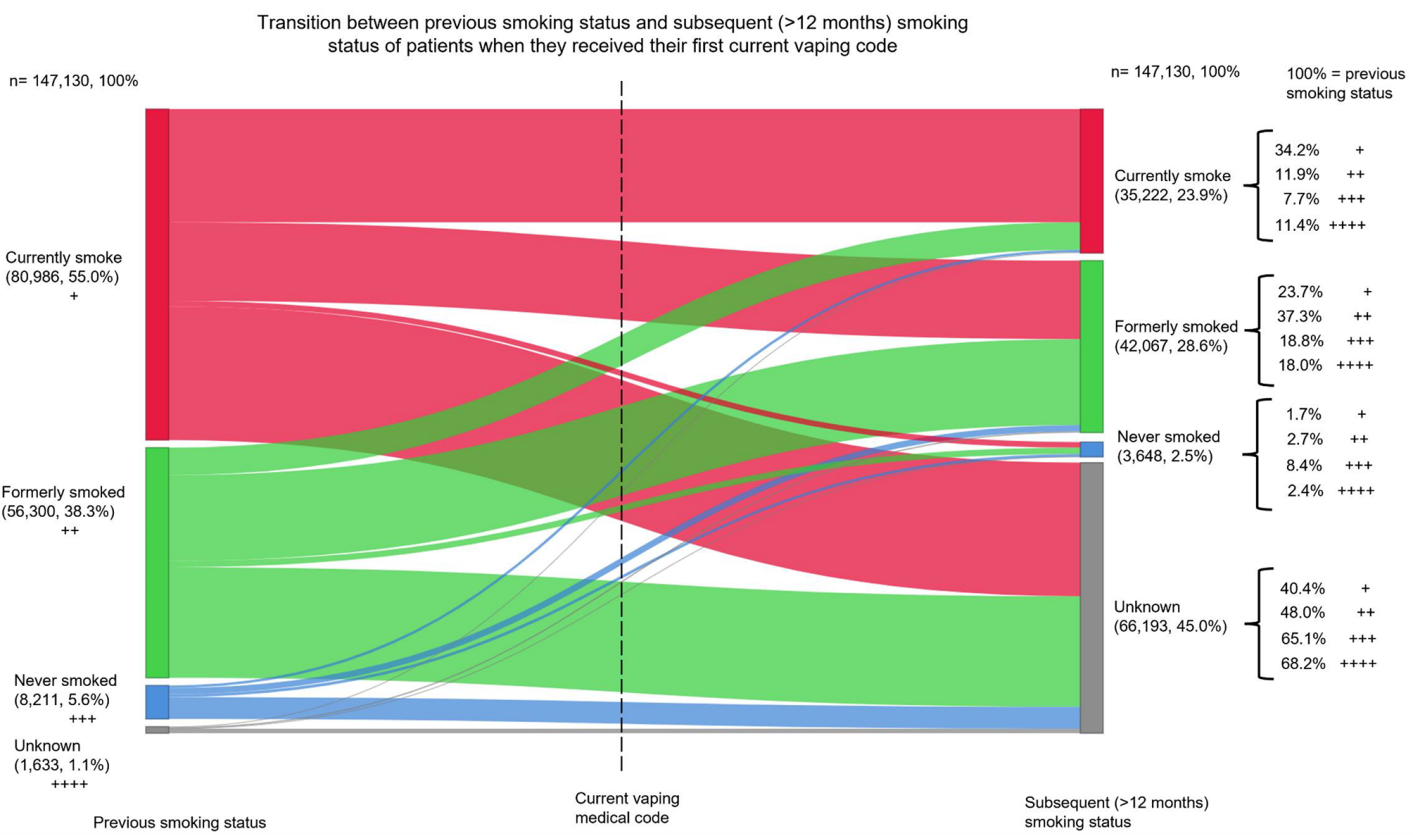
Transition between previous smoking status and subsequent (> 12 months) smoking status of patients when they received their first current vaping code The ‘nodes’ (vertical bars) are coloured to represent the smoking status record obtained in the consultation (red: currently smoke, green: formerly smoked, blue: never smoked, unknown: grey). The ‘connections’ (transitions from left to right) are coloured to represent the previous smoking status (red: currently smoke, green: formerly smoked, blue: never smoked, unknown: grey) The + signs on the right side (subsequent smoking status) indicate the proportion breakdown of previous smoking status categories. For example: Those who ‘currently smoke’ before receiving the current vaping code, > 12 months after they received the current vaping code: 34.2% of them were currently smoking, 23.7% of them had quit smoking, 1.7% received a ‘never smoked’ code, and 40.4% had no smoking status recorded. (34.2% ‘ 23.7% ‘ 1.7% ‘ 40.4% = 100%) The mean time difference between the previous smoking status record and the current vaping medical code record was 542.6 days (SD: 668.1 days, range: 1.0 to 14,729.0, median: 344.0). The mean time difference between the subsequent smoking status record and the current vaping medical code record was 1,180.0 days (SD: 561.8, range: 366.0 to 3,372.0, median: 1,085.0)

Of all patients who received a first-time *current* vaping code, 98.9% (145,497/147,130) had a previous current smoking status recording. The majority were smoking (n = 80,986, 55.0%) or formerly smoked (n = 56,300, 38.3%) before receiving the vaping code, while 5.6% (n = 8,211) of patients had never smoked (Fig. 5).

Over half (80,937/147,130, 55.0%) of patients had a subsequent current smoking status recording.

Over a year after receiving the initial *current* vaping code, over a third (34.2%) of people who were smoking before they received the vaping code were still smoking, just under a quarter (23.7%) were indicated to have quit smoking, 1.7% received a ‘never smoked’ status, and there was no smoking status record for 40.4%.

Over a year after receiving the initial *current* vaping code, 11.9% of people who had quit smoking before they received the vaping code had returned to smoking, over a third (37.3%) were indicated to still be quit smoking, 2.7% received a ‘never smoked’ status, and there was no smoking status record for 48.0%.

Over a year after receiving the initial *current* vaping code, 7.7% of people who had never smoked before they received the vaping code had initiated smoking, 18.8% were indicated to have quit smoking, 8.4% still had a ‘never smoked’ status, and there was no smoking status record for 65.1%.

##### Former vaping

Out of all patients who received for the first-time a *former* vaping code, 99.3% (5,110/5,147) had a previous current smoking status recording and 60.6% (3,121/5,147) had a subsequent current smoking status recording (Supplementary Graph 2).

See Supplementary Tables 5a and 5b for additional detail. Results from the sensitivity analysis (Supplementary Graph 3) where the subsequent smoking status was recorded between > 12 months to ≤ 24 months after receiving the first-time current vaping code were similar to the main analysis (Fig. 5).

## Discussion

Using 2006–2022 CPRD UK primary care data, we identified seven medical codes indicating current or former vaping. Vaping code incidence increased from September 2013. The EVALI outbreak in the US (and peak media coverage in September 2019) was significantly associated with a reduction in new records of current vaping, manifested as a declining trend over a period of seven months (September 2019 to March 2020); additionally, there was an immediate increase in new records of former vaping, followed by a declining trend over the subsequent seven-month period. When patients received their first vaping code, mean age was 50.2 years, 52.4% were female, and 82.1% were White. When receiving the first vaping code, the majority of patients were either smoking or had quit smoking in the past, and < 2% were recorded as having never smoked. Of those recorded as currently vaping, 98.9% had records of their previous smoking status, and 55.0% had records of their > 12 months smoking status. Over a year after being recorded as vaping, 34.2% of people who were smoking prior to being recorded as vaping were still smoking, 23.7% quit smoking, 1.7% received a ‘never smoked’ status, and there was no smoking status for 40.4%.

Similar to US studies [10–14, 16, 17], we found that vaping documentation incidence in UK EHRs is low, but has increased over time. We found no medical codes indicating never vaping. There was a rising trend in new current and former vaping records over time, this may be attributed to an increase in: awareness of vaping or the relevant vaping codes among GPs; GPs screening the vaping status of their patients; or patients volunteering their vaping status or having questions about their vaping status to GPs.

The changes associated with the EVALI outbreak could be attributable to increasing numbers of patients quitting vaping due to negative media coverage of potential health harms or GPs paying greater attention to asking and recording about (former) vaping. To our knowledge, no other study has examined the effect of EVALI on vaping documentation in EHRs.

The reduction in monthly number of new current and vaping records following implementation of the first national COVID-19 lockdown could be attributable to reduced access to GP appointments.

Unlike US studies [14, 15], where patients with vaping documentation were more likely to be younger, the mean age of patients in our sample when they first received a vaping code was 50 years. A 2022 Great Britain vaping survey [31] found that 11% of 18–44-year-olds, and 10% of 45–55-year-olds used NVPs, indicating that a relatively high proportion of middle-aged people use NVPs. Our finding may reflect the NVP prevalence in Great Britain, that we excluded patients < 18 years, and that older people may be more likely to visit a health professional, and hence have more opportunities to receive a vaping code.

The gender distribution in our sample of patients who have ever received a vaping medical code was similar to the 2021 England and Wales Census [32] (51.0% female). However, Great Britain vaping surveys [31] found that a higher proportion of males use NVPs compared to females, similar to two US studies [14, 15].

Similar to US studies [14, 15], we found that most patients who have ever received a vaping code were White (82.1%), reflecting UK population ethnicity proportions [33]. However, our other ethnicity categories were confounded by 12.6% being ‘unknown’ (similar to a previous CPRD study [26]).

Our findings are similar to previous studies where a high proportion of those with vaping documentation were currently smoking (57% [14, 16], 52.4% [17]) or formerly smoked (35% [14, 16]).

Two US studies found that among those who smoked and vaped, 20.8% [15] and 23.0% [16] reported quitting smoking during the following year. Our finding was similar: > 12 months after receiving a current vaping code, 23.7% of people who were smoking before they received the vaping code were indicated to have quit smoking. Additionally, Young-Wolff et al. [16] found that among those who quit smoking before vaping, 14.0% of those currently vaping reported returning to smoking in the following year; we found that 11.9% of people who had quit smoking before they received the vaping code had returned to smoking after 12 months. Lastly, we found that among those who have never smoked before they received the vaping code, 7.7% had initiated smoking after > 12 months after receiving the current vaping code, compared with 8.0% in the prior study [16]. However, we cannot make inferences about the effectiveness of NVP use on smoking cessation from our analyses because ~ 45% of patients did not have a > 12-month follow-up smoking status record, vaping documentation is likely to be missing not at random, and we did not control for any confounding factors.

### Strengths & limitations

Our study has several strengths. This is the first study to comprehensively describe and characterise NVP use recording in UK EHRs. We used data from CPRD which covers 25% of the UK population. Our study covers 16 years from when NVPs appeared in England in 2006 to March 2022. We found that CPRD vaping record data were sufficiently sensitive to be able to detect statistically significant effects of events (EVALI, COVID-19 lockdown) on vaping record incidence.

Our study also has limitations. We could not analyse free-text comments that GPs can log, as these are not available for research purposes. While CPRD data have been shown to be largely representative of the UK population [25–27], CPRD may be less representative for specific subgroups, such as people who vape. Vaping status and smoking status may not be accurately captured in EHRs, e.g., some patients were recorded to be smoking or have quit smoking before receiving a vaping code, but they received a ‘never smoked’ record > 12 months after. In our smoking status transition analyses, the time between the vaping code consultation date and the subsequent smoking status consultation date varied between patients because we wanted to capture the longest possible smoking cessation outcome for each patient. Given that smoking is a relapsing and remitting condition, the variable duration of the follow-up record may limit the interpretation of our results, however, we conducted a sensitivity analysis to mitigate this. The interpretation of the smoking status transitions is limited regarding the smoking cessation rate following receiving a vaping code because a high proportion of patients (44.8%, 68,219/152,277) did not have a subsequent smoking status recording.

### Implications

Vaping documentation rates in primary care UK EHRs are very low. Increased completeness, accuracy and consistency of vaping status recording would further increase the value of these data. Refining existing medical codes would enable health professionals to unambiguously record current vaping, former vaping or never vaping. Financially incentivising health professionals has increased smoking status recording [34]; in the UK, a QOF indicator could be introduced for recording vaping status. In clinical practice, vaping screening could be assigned to specific clinical team members [13] or integrated into existing processes, such as alongside routine smoking screening during annual health checks [13].

Improving the completeness of EHR vaping status data would result in longitudinal population-level data for vaping surveillance which is linkable to other electronic health information. This could be employed to investigate long-term health outcomes of vaping [14, 23], evidence on which is currently lacking. We found that nearly all first-time incidence of vaping records had a previous smoking status recording, and more than half had a subsequent smoking status recording. Future studies could employ matched control samples to investigate if there are any differences between longer-term smoking cessation outcomes [15, 16] or health outcomes between patients who vape and do not vape. Also, studies using EHRs could investigate how long patients use NVPs, and transitions between current and former vaping and vice versa.

## Conclusion

Using 2006–2022 CPRD UK primary care data, we found that vaping code incidence increased from September 2013 but vaping documentation rates were overall very low. When receiving the first vaping code, the majority of patients were either smoking or had quit smoking in the past. Of those who were smoking prior to being recorded as currently vaping, more than a year after the vaping record, over a third were still smoking, under a quarter quit smoking, and there was no follow-up smoking status record for 40%. Increased completeness, accuracy and consistency of vaping status recording would further increase the value of longitudinal population-level EHR data, enabling the investigation of the long-term health effects of vaping.

### Electronic supplementary material

Below is the link to the electronic supplementary material.

Supplementary Material 1

## Data Availability

Source data for this study were obtained from the Clinical Practice Research Datalink (CPRD). These data sources are made available for scientific and medical research after submission of a study protocol to be reviewed and approved by the CPRD Independent Scientific Advisory Committee (ISAC). Owing to ethical restrictions, the data used in this analysis are not publicly available, in line with the data privacy rules set up by CPRD/ISAC. Data access queries can be directed to enquiries@cprd.com.

## List of Abbreviations

CPRD: Clinical Practice Research Datalink
EHR: Electronic health records
EVALI: e-cigarette or vaping product use-associated lung injury
GP: General practitioner
NVPs: Nicotine vaping products (or e-cigarettes typically used with nicotine)
UK: United Kingdom
US: United States
QOF: Quality and Outcomes Framework
