# Supplementary material for: How is nicotine vaping product (e-cigarette) use monitored in primary care electronic health records in the United Kingdom? An exploratory analysis of Clinical Practice Research Datalink (CPRD)
Source: BMC Public Health. 2023 Nov 16;23:2263. doi: 10.1186/s12889-023-17200-7 (PMC10655457; doi:10.1186/s12889-023-17200-7)

**How is nicotine vaping product (e-cigarette) use monitored in primary care electronic health records in the United Kingdom? An exploratory analysis of the Clinical Practice Research Datalink (CPRD).**

Supplementary Material

Contents

[Supplementary Box 1. Relevant studies identified by systematic literature search 2](#_Toc148455921)

[Supplementary Box 2. CPRD Denominator files 3](#_Toc148455922)

[Supplementary Figure 1. Study inclusion – observations of vaping codes from 1 September 2006 to 31 March 2022 4](#_Toc148455923)

[Supplementary Figure 2. Illustration of patient-level first-time incidence of vaping codes samples 5](#_Toc148455924)

[Supplementary Table 1. Vaping medical codes in CPRD 6](#_Toc148455925)

[Supplementary Table 2a. Ethnicity medical codes – CPRD GOLD 6](#_Toc148455926)

[Supplementary Table 2b. Ethnicity medical codes – CPRD Aurum 12](#_Toc148455927)

[Supplementary Table 3a. Smoking status medical codes – CPRD GOLD 19](#_Toc148455928)

[Supplementary Table 3b. Smoking status medical codes – CPRD Aurum 20](#_Toc148455929)

[Supplementary Table 4. Frequency of vaping codes per unique patient 25](#_Toc148455930)

[Supplementary Table 5a. Previous and subsequent smoking status of patients who received a current vaping code 26](#_Toc148455931)

[Supplementary Table 5b. Previous and subsequent smoking status of patients who received a former vaping code 27](#_Toc148455932)

[Supplementary Graph 1: Patient-level first-time incidence of current vaping and former medical codes, by region 28](#_Toc148455933)

[Supplementary Graph 2: Transition between previous smoking status and subsequent (>12 months) smoking status of patients when they received their first former vaping code 29](#_Toc148455934)

[Supplementary Graph 3: Transition between previous smoking status and subsequent (>12–≤24 months) smoking status of patients when they received their first current vaping code 31](#_Toc148455935)

## Supplementary Box 1. Relevant studies identified by systematic literature search

We conducted a systematic literature search of MEDLINE (OVID) for articles published from 1946 to 8 August 2023 using the following subject headings and key words: (“e-cigarette$” OR “electronic nicotine delivery system$” OR “vaping” OR “vape$” OR “electronic cigarette$”) AND (“electronic health$” OR “electronic medical” OR “electronic patient$” OR “medic$ info$ system$”). The search yielded 36 records of which 13 were relevant. All studies were from the United States.

D’Angelo, H., Land, S.R. and Mayne, R.G. (2021) ‘Assessing Electronic Nicotine Delivery Systems Use at NCI-Designated Cancer Centers in the Cancer Moonshot-funded Cancer Center Cessation Initiative’, *Cancer prevention research (Philadelphia, Pa.)*, 14(8), pp. 763–766. Available at: https://doi.org/10.1158/1940-6207.CAPR-21-0105.

Heiden, B.T. *et al.* (2022) ‘Assessment of formal tobacco treatment and smoking cessation in dual users of cigarettes and e-cigarettes’, *Thorax*, 78(3), pp. 267–273. Available at: https://doi.org/10.1136/THORAX-2022-218680.

Hurst, S. and Conway, M. (2018) ‘Exploring Physician Attitudes Regarding Electronic Documentation of E-cigarette Use: A Qualitative Study’, *Tobacco use insights*, 11, pp. 1179173X18782879-1179173X18782879. Available at: https://doi.org/10.1177/1179173X18782879.

Jose, T., Hays, J.T. and Warner, D.O. (2020) ‘Improved Documentation of Electronic Cigarette Use in an Electronic Health Record’, *International journal of environmental research and public health*, 17(16), p. 5908. Available at: https://doi.org/10.3390/ijerph17165908.

Khanna, N. *et al.* (2023) ‘Integrating a Systematic, Comprehensive E-Cigarette and Vaping Assessment Tool into the Electronic Health Record’, *The Journal of the American Board of Family Medicine*, 36(3), pp. 405–413. Available at: https://doi.org/10.3122/JABFM.2022.220410R1.

Kovach, K.A. *et al.* (2021) ‘Co-creating opportunities to incorporate cessation for electronic nicotine delivery systems in family medicine - a qualitative program evaluation’, *BMC family practice*, 22(1). Available at: https://doi.org/10.1186/S12875-021-01520-X.

LeLaurin, J.H. *et al.* (2020) ‘Tobacco-Related Counseling and Documentation in Adolescent Primary Care Practice: Challenges and Opportunities’, *Nicotine & tobacco research : official journal of the Society for Research on Nicotine and Tobacco*, 22(6), pp. 1023–1029. Available at: https://doi.org/10.1093/ntr/ntz076.

Rodriguez, Z.C. *et al.* (2021) ‘Vaping: Impact of Improving Screening Questioning in Adolescent Population: A Quality Improvement Initiative’, *Pediatric Quality & Safety*, 6(1), p. e370. Available at: https://doi.org/10.1097/PQ9.0000000000000370.

Sanford, B.T. *et al.* (2023) ‘E-Cigarette Screening in Primary Care’, *American Journal of Preventive Medicine* [Preprint]. Available at: https://doi.org/10.1016/J.AMEPRE.2023.02.030.

Winden, T.J. *et al.* (2015) ‘Towards the Standardized Documentation of E-Cigarette Use in the Electronic Health Record for Population Health Surveillance and Research’, *AMIA Joint Summits on Translational Science*, 2015, pp. 199–203.

Young-Wolff, K.C. *et al.* (2017) ‘Do you vape? Leveraging electronic health records to assess clinician documentation of electronic nicotine delivery system use among adolescents and adults’, *Preventive medicine*, 105, p. 32. Available at: https://doi.org/10.1016/J.YPMED.2017.08.009.

Young-Wolff, K.C. *et al.* (2018) ‘Documentation of e-cigarette use and associations with smoking from 2012 to 2015 in an integrated healthcare delivery system’, *Preventive medicine*, 109, p. 113. Available at: https://doi.org/10.1016/J.YPMED.2018.01.012.

Young-Wolff, K.C. *et al.* (2022) ‘Electronic cigarette use and risk of COVID-19 among young adults without a history of cigarette smoking’, *Preventive medicine*, 162. Available at: https://doi.org/10.1016/J.YPMED.2022.107151.

## Supplementary Box 2. CPRD Denominator files

As new GP practices join and contribute their (historical and ongoing) patient data to CPRD, denominator files can be used to calculate the number of patients contributing to CPRD at specific time periods, which can be used to calculate point prevalence or incidence rate.

After applying the study exclusion criteria to the denominator files, the number of patients (aged ≥18 years) contributing data to CPRD in each month from September 2011 to March 2022 was calculated.

The ‘start date’ for each patient contributing to CPRD was the chronologically latest of ‘registration start date’ and ‘current registration date’; the ‘end date’ for each patient contributing to CPRD was the chronologically earliest of the ‘registration end date’, ‘death date’, ‘transfer out date’, or ‘last collection date’ of the practice.

## Supplementary Figure 1. Study inclusion – observations of vaping codes from 1 September 2006 to 31 March 2022

Medical code exclusions (n= 57)

n= 1 observations of “e-cigarette” code

n= 55 observations of “Electronic cigarette” code

n= 1 observations of “Electronic cigarette liquid” code

CPRD GOLD

Build: April 2023

n= 7,915 observations of vaping codes

n= 6,345 unique patients

CPRD Aurum

Build: March 2023

n= 221,848 observations of medical codes

n= 145,481 unique patients

CPRD Aurum exclusions (n= 3,666 observations in total)

n = 1,156 observations which had a practice ID which was recommended for exclusion

n = 112 observations where the patient was aged <18 years

n = 2,412 duplicated observations

CPRD GOLD

n= 6,986 observations of vaping codes

n= 5,511 unique patients

CPRD Aurum

n=218,182 observations of vaping codes

n=144,627 unique patients

CPRD GOLD exclusions (n=929 observations in total)

n = 4 observations where the patient was aged <18 years

n = 39 duplicated observations

n = 896 observations from practices which have migrated to CPRD Aurum

CPRD GOLD and CPRD Aurum merged

n= 225,168 observations of vaping codes

n= 150,138 unique patients

Analytical dataset

n= 225,111 observations of current or former vaping codes

n= 150,114 unique patients

## Supplementary Figure 2. Illustration of patient-level first-time incidence of vaping codes samples

Current vaping code

Former vaping code

Both Current vaping code and Former vaping code

n= 144,967 unique patients

n= 2,984
unique patients

n= 2,163
unique patients

n= 150,144 unique patients

n= 147,130 observations of first-time Current vaping codes

n= 5,147 observations of first-time Former vaping codes

n= 152,277 observations of first-time incidence of vaping codes

(147,130 + 5,147 = 152,277)

## Supplementary Table 1. Vaping medical codes in CPRD

| **Medical code terms** | **Dataset** | **Medical code** |
| --- | --- | --- |
| User of electronic cigarette | GOLD | 107292 |
| Ex user of electronic cigarette | GOLD | 108503 |
| e-cigarette user | Aurum | 7832561000006114 |
| Electronic cigarette user | Aurum | 2265711000000117 |
| User of electronic cigarette | Aurum | 1879431000006110 |
| Vaper with nicotine | Aurum | 13653501000006119 |
| Ex user of electronic cigarette | Aurum | 2336091000000117 |
| e-cigarette | Aurum | 7832541000006110 |
| Electronic cigarette | Aurum | 7832521000006115 |
| Electronic cigarette liquid | Aurum | 3513803011 |

Using the CPRD medical code browser, medical code terms related to electronic cigarettes/e-cigarettes, vaping/vaper, electronic nicotine delivery systems (ENDS), and e-liquid were identified. Non-specific codes which only related to “nicotine user” or “nicotine dependent/dependence” were excluded.

## Supplementary Table 2a. Ethnicity medical codes – CPRD GOLD

| **Medical code** | **Read Term** | **Ethnicity** |
| --- | --- | --- |
| 47005 | Asian and Chinese - ethnic category 2001 census | Asian |
| 110422 | Asian or Asian British: Indian - NI ethnic cat 2011 census | Asian |
| 110590 | Asian/Asian Brit: Bangladeshi- Eng+Wales eth cat 2011 census | Asian |
| 110922 | Asian/Asian Brit: Chinese - Eng+Wales ethnic cat 2011 census | Asian |
| 110477 | Asian/Asian Brit: Indian - Eng+Wales ethnic cat 2011 census | Asian |
| 111743 | Asian/Asian Brit: other Asian- Eng+Wales eth cat 2011 census | Asian |
| 110720 | Asian/Asian British: Bangladeshi - NI ethnic cat 2011 census | Asian |
| 112363 | Asian/Asian British: Chinese - NI ethnic cat 2011 census | Asian |
| 110425 | Asian/Asian British: other Asian - NI ethnic cat 2011 census | Asian |
| 110538 | Asian/Asian British: Pakistani - NI ethnic cat 2011 census | Asian |
| 110464 | Asian/Asian British:Pakistani- Eng+Wales eth cat 2011 census | Asian |
| 111064 | Asian: Chinese - Scotland ethnic category 2011 census | Asian |
| 111368 | Asian: Indian, Indian Scot/Indian Brit- Scotland 2011 census | Asian |
| 110855 | Asian: other Asian group - Scotland ethnic cat 2011 census | Asian |
| 110460 | Asian: Pakistani/Pakistani Scot/Pakistani Brit- Scot 2011 | Asian |
| 24740 | Bangladeshi | Asian |
| 28888 | Bangladeshi or British Bangladeshi - ethn categ 2001 census | Asian |
| 112225 | Bangladeshi, Bangladeshi Scot or Bangladeshi Brit- Scot 2011 | Asian |
| 12653 | British Asian - ethnic category 2001 census | Asian |
| 63872 | Buddhist - ethnic category 2001 census | Asian |
| 24272 | Chinese | Asian |
| 12718 | Chinese | Asian |
| 12468 | Chinese - ethnic category 2001 census | Asian |
| 38097 | E Afric Asian/Indo-Carib (NMO) | Asian |
| 47077 | East African Asian - ethnic category 2001 census | Asian |
| 46818 | East African Asian (NMO) | Asian |
| 12420 | Filipino - ethnic category 2001 census | Asian |
| 56127 | Hindu - ethnic category 2001 census | Asian |
| 25920 | Indian | Asian |
| 12482 | Indian | Asian |
| 12414 | Indian or British Indian - ethnic category 2001 census | Asian |
| 12473 | Japanese - ethnic category 2001 census | Asian |
| 64133 | Kashmiri - ethnic category 2001 census | Asian |
| 12730 | Malaysian - ethnic category 2001 census | Asian |
| 46056 | Mixed Asian - ethnic category 2001 census | Asian |
| 32396 | Other Asian | Asian |
| 26379 | Other Asian (NMO) | Asian |
| 12513 | Other Asian background - ethnic category 2001 census | Asian |
| 12668 | Other Asian ethnic group | Asian |
| 28935 | Other Asian or Asian unspecified ethnic category 2001 census | Asian |
| 32401 | Other ethnic, Asian/White orig | Asian |
| 24690 | Pakistani | Asian |
| 12460 | Pakistani or British Pakistani - ethnic category 2001 census | Asian |
| 26392 | Punjabi - ethnic category 2001 census | Asian |
| 49658 | Sikh - ethnic category 2001 census | Asian |
| 12887 | Sinhalese - ethnic category 2001 census | Asian |
| 46649 | South East Asian | Asian |
| 12608 | Sri Lankan - ethnic category 2001 census | Asian |
| 12760 | Tamil - ethnic category 2001 census | Asian |
| 25411 | Vietnamese | Asian |
| 12719 | Vietnamese - ethnic category 2001 census | Asian |
| 12350 | African - ethnic category 2001 census | Black |
| 111059 | African: African/African Scot/African Brit - Scotland 2011 | Black |
| 110655 | African: any other African - Scotland ethnic cat 2011 census | Black |
| 35412 | Black - other African country | Black |
| 35350 | Black - other Asian | Black |
| 25676 | Black - other, mixed | Black |
| 12778 | Black African | Black |
| 32443 | Black African and White | Black |
| 12795 | Black and Asian - ethnic category 2001 census | Black |
| 49940 | Black and Chinese - ethnic category 2001 census | Black |
| 40110 | Black and White - ethnic category 2001 census | Black |
| 57752 | Black Arab | Black |
| 26312 | Black Black - other | Black |
| 12452 | Black British | Black |
| 40097 | Black British - ethnic category 2001 census | Black |
| 47950 | Black Caribbean | Black |
| 12632 | Black Caribbean | Black |
| 32425 | Black Caribbean and White | Black |
| 57435 | Black Caribbean/W.I./Guyana | Black |
| 47965 | Black E Afric Asia/Indo-Caribb | Black |
| 57753 | Black East African Asian | Black |
| 32100 | Black Guyana | Black |
| 48005 | Black Indian sub-continent | Black |
| 57763 | Black Indo-Caribbean | Black |
| 50286 | Black Iranian | Black |
| 41329 | Black N African/Arab/Iranian | Black |
| 46812 | Black North African | Black |
| 47997 | Black West Indian | Black |
| 24339 | Black, other, non-mixed origin | Black |
| 110540 | Black/Afr/Carib/Black Brit: other Black- Eng+Wales 2011 cens | Black |
| 110630 | Black/Afri/Carib/Black Brit: African- NI eth cat 2011 census | Black |
| 110779 | Black/Afri/Carib/Black Brit: Caribbean- NI eth cat 2011 cens | Black |
| 111880 | Black/Afri/Carib/Black Brit: other - NI eth cat 2011 census | Black |
| 110437 | Black/African/Carib/Black Brit: African- Eng+Wales 2011 cens | Black |
| 110436 | Black/African/Caribbn/Black Brit: Caribbean - Eng+Wales 2011 | Black |
| 112216 | Carib/Black: any other Black/Caribbean grp - Scotland 2011 | Black |
| 112649 | Carib/Black: Black/Black Scot/Black Brit- Scotland 2011 cens | Black |
| 113671 | Carib/Black: Caribbean/Carib Scot/Carib Brit- Scotland 2011 | Black |
| 12432 | Caribbean - ethnic category 2001 census | Black |
| 32399 | Caribbean Asian - ethnic category 2001 census | Black |
| 54593 | Caribbean I./W.I./Guyana (NMO) | Black |
| 57094 | Caribbean Island (NMO) | Black |
| 99316 | Indo-Caribbean (NMO) | Black |
| 40096 | Mixed Black - ethnic category 2001 census | Black |
| 32886 | Nigerian - ethnic category 2001 census | Black |
| 47028 | North African - ethnic category 2001 census | Black |
| 32165 | Other Black - Black/Asian orig | Black |
| 25623 | Other Black - Black/White orig | Black |
| 32389 | Other Black background - ethnic category 2001 census | Black |
| 32136 | Other black ethnic group | Black |
| 46047 | Other Black or Black unspecified ethnic category 2001 census | Black |
| 46752 | Other Pacific ethnic group | Black |
| 12443 | Somali - ethnic category 2001 census | Black |
| 57075 | West Indian (NMO) | Black |
| 12706 | Chinese and White - ethnic category 2001 census | Mixed |
| 110696 | Mixed/multiple ethnic grps: any- Scot ethnic cat 2011 census | Mixed |
| 110654 | Mixed: other Mixed/multiple backgrd - Eng+Wales 2011 census | Mixed |
| 110536 | Mixed: other Mixed/multiple ethnic backgrd - NI 2011 census | Mixed |
| 110471 | Mixed: White and Asian - NI ethnic category 2011 census | Mixed |
| 110651 | Mixed: White and Black African - NI ethnic cat 2011 census | Mixed |
| 110661 | Mixed: White and Black Caribbean - NI ethnic cat 2011 census | Mixed |
| 110652 | Mixed: White+Asian - Eng+Wales ethnic category 2011 census | Mixed |
| 110421 | Mixed: White+Black African - Eng+Wales eth cat 2011 census | Mixed |
| 110445 | Mixed: White+Black Caribbean - Eng+Wales eth cat 2011 census | Mixed |
| 47401 | Other ethnic, Black/White orig | Mixed |
| 12696 | Other ethnic, mixed origin | Mixed |
| 32420 | Other ethnic, other mixed orig | Mixed |
| 12873 | Other Mixed background - ethnic category 2001 census | Mixed |
| 32408 | Other Mixed or Mixed unspecified ethnic category 2001 census | Mixed |
| 12638 | White and Asian - ethnic category 2001 census | Mixed |
| 12437 | White and Black African - ethnic category 2001 census | Mixed |
| 12742 | White and Black Caribbean - ethnic category 2001 census | Mixed |
| 26455 | Any other group - ethnic category 2001 census | Other |
| 46059 | Arab - ethnic category 2001 census | Other |
| 32110 | Brit. ethnic minor. spec.(NMO) | Other |
| 57764 | Brit. ethnic minor. unsp (NMO) | Other |
| 12435 | Ethnic category - 2001 census | Other |
| 110472 | Ethnic category - 2011 census | Other |
| 110417 | Ethnic category - 2011 census England and Wales | Other |
| 112302 | Ethnic category - 2011 census Northern Ireland | Other |
| 110962 | Ethnic category - 2011 census Scotland | Other |
| 12459 | Ethnic category not stated - 2001 census | Other |
| 10196 | Ethnic groups (census) | Other |
| 45199 | Ethnic groups (census) NOS | Other |
| 23955 | Ethnicity and other related nationality data | Other |
| 64609 | Fijian | Other |
| 25937 | Iranian - ethnic category 2001 census | Other |
| 25082 | Iranian (NMO) | Other |
| 45964 | Kurdish - ethnic category 2001 census | Other |
| 26246 | Latin American - ethnic category 2001 census | Other |
| 25451 | Moroccan - ethnic category 2001 census | Other |
| 47091 | Muslim - ethnic category 2001 census | Other |
| 24962 | N African Arab/Iranian (NMO) | Other |
| 47285 | North African Arab (NMO) | Other |
| 25969 | O/E - ethnic group | Other |
| 60284 | O/E - ethnic group NOS | Other |
| 12332 | O/E - ethnic origin | Other |
| 12434 | Other - ethnic category 2001 census | Other |
| 12757 | Other ethnic group | Other |
| 110646 | Other ethnic group: any other grp- NI ethnic cat 2011 census | Other |
| 110555 | Other ethnic group: Arab - Eng+Wales ethnic cat 2011 census | Other |
| 110780 | Other ethnic group: Arab - NI ethnic category 2011 census | Other |
| 111806 | Other ethnic grp: any other ethnic grp- Scotland 2011 census | Other |
| 112245 | Other ethnic grp: Arab/Arab Scot/Arab British- Scotland 2011 | Other |
| 41214 | Other ethnic NEC (NMO) | Other |
| 30280 | Other ethnic non-mixed (NMO) | Other |
| 110742 | Other ethnic: any other grp - Eng+Wales eth cat 2011 census | Other |
| 64610 | Samoan | Other |
| 12756 | South and Central American - ethnic category 2001 census | Other |
| 94487 | Yemeni | Other |
| 25422 | Albanian - ethnic category 2001 census | White |
| 12433 | Baltic Estonian/Latvian/Lithuanian - ethn categ 2001 census | White |
| 46956 | Bosnian - ethnic category 2001 census | White |
| 12351 | British or mixed British - ethnic category 2001 census | White |
| 99788 | Bulgarian | White |
| 28887 | Cornish - ethnic category 2001 census | White |
| 28866 | Croatian - ethnic category 2001 census | White |
| 32778 | Cypriot (part not stated) - ethnic category 2001 census | White |
| 100143 | Czech | White |
| 12352 | English - ethnic category 2001 census | White |
| 12355 | Greek - ethnic category 2001 census | White |
| 45955 | Greek (NMO) | White |
| 12769 | Greek Cypriot - ethnic category 2001 census | White |
| 47949 | Greek Cypriot (NMO) | White |
| 45947 | Greek/Greek Cypriot (NMO) | White |
| 42290 | Gypsy/Romany - ethnic category 2001 census | White |
| 12532 | Irish - ethnic category 2001 census | White |
| 24270 | Irish (NMO) | White |
| 47601 | Irish traveller | White |
| 55223 | Irish Traveller - ethnic category 2001 census | White |
| 115519 | Irish Traveller - Northern Ireland ethnic cat 2011 census | White |
| 46964 | Israeli - ethnic category 2001 census | White |
| 12412 | Italian - ethnic category 2001 census | White |
| 46063 | Jewish - ethnic category 2001 census | White |
| 26341 | Kosovan - ethnic category 2001 census | White |
| 26391 | Mixed Irish and other White - ethnic category 2001 census | White |
| 71425 | New Zealand ethnic group NOS | White |
| 45008 | New Zealand ethnic groups | White |
| 57286 | New Zealand European | White |
| 32479 | New Zealand Maori | White |
| 42294 | Northern Irish - ethnic category 2001 census | White |
| 12402 | Oth White European/European unsp/Mixed European 2001 census | White |
| 35459 | Other ethnic, mixed white orig | White |
| 12633 | Other European (NMO) | White |
| 85505 | Other European in New Zealand | White |
| 28900 | Other mixed White - ethnic category 2001 census | White |
| 96789 | Other New Zealand ethnic group | White |
| 28936 | Other republics former Yugoslavia - ethnic categ 2001 census | White |
| 12421 | Other White background - ethnic category 2001 census | White |
| 26310 | Other white British ethnic group | White |
| 12444 | Other white ethnic group | White |
| 12591 | Other White or White unspecified ethnic category 2001 census | White |
| 12467 | Polish - ethnic category 2001 census | White |
| 101219 | Portuguese | White |
| 99808 | Romanian | White |
| 12436 | Scottish - ethnic category 2001 census | White |
| 47074 | Serbian - ethnic category 2001 census | White |
| 55113 | Traveller - ethnic category 2001 census | White |
| 12746 | Turkish - ethnic category 2001 census | White |
| 32126 | Turkish (NMO) | White |
| 32413 | Turkish Cypriot - ethnic category 2001 census | White |
| 32069 | Turkish Cypriot (NMO) | White |
| 32066 | Turkish/Turkish Cypriot (NMO) | White |
| 40102 | Ulster Scots - ethnic category 2001 census | White |
| 12681 | Welsh - ethnic category 2001 census | White |
| 22467 | White | White |
| 112899 | White - Northern Ireland ethnic category 2011 census | White |
| 12446 | White British | White |
| 98111 | White British - ethnic category 2001 census | White |
| 24837 | White Irish | White |
| 98213 | White Irish - ethnic category 2001 census | White |
| 26467 | White Scottish | White |
| 111386 | White: Gypsy/Irish Traveller - Eng+Wales eth cat 2011 census | White |
| 113253 | White: Gypsy/Irish Traveller - Scotland ethnic cat 2011 cens | White |
| 110556 | White: Irish - England and Wales ethnic category 2011 census | White |
| 110687 | White: Irish - Scotland ethnic category 2011 census | White |
| 110694 | White: other British - Scotland ethnic category 2011 census | White |
| 110407 | White: other White backgrd- Eng+Wales ethnic cat 2011 census | White |
| 110695 | White: other White ethnic grp- Scotland ethnic cat 2011 cens | White |
| 110465 | White: Polish - Scotland ethnic category 2011 census | White |
| 110432 | White: Scottish - Scotland ethnic category 2011 census | White |
| 110420 | White:Eng/Welsh/Scot/NI/Brit - England and Wales 2011 census | White |

Where a patient had inconsistent records for ethnicity, the most frequently occurring category for that patient was used. Ethnicity was recorded as ‘unknown’ if no category was most frequent or if no information was recorded.

## Supplementary Table 2b. Ethnicity medical codes – CPRD Aurum

| **Medical code** | **Term** | **Ethnicity** |
| --- | --- | --- |
| 141531000000112 | Kashmiri - ethnic category 2001 census | Asian |
| 1564521000006118 | Chinese | Asian |
| 1968481000006118 | Asian or Asian Scottish or Asian British: Pakistani, Pakistani Scottish or Pakistani British - Scotland ethnic category 2011 census | Asian |
| 550541000006110 | Chinese | Asian |
| 141651000000118 | Malaysian - ethnic category 2001 census | Asian |
| 1564921000006112 | Indian | Asian |
| 285977016 | Other Asian (NMO) | Asian |
| 216045018 | Reads Chinese | Asian |
| 411583012 | East African Asian (NMO) | Asian |
| 781081000006113 | Indian | Asian |
| 141361000000114 | Pakistani or British Pakistani - ethnic category 2001 census | Asian |
| 285956019 | Bangladeshi | Asian |
| 141641000000116 | Filipino - ethnic category 2001 census | Asian |
| 285955015 | Pakistani | Asian |
| 1968171000006110 | Asian or Asian British: any other Asian background - England and Wales ethnic category 2011 census | Asian |
| 1968131000006112 | Asian or Asian British: Indian - England and Wales ethnic category 2011 census | Asian |
| 285954016 | Indian | Asian |
| 136081000000111 | Sri Lankan - ethnic category 2001 census | Asian |
| 1565441000006111 | Pakistani | Asian |
| 141561000000119 | British Asian - ethnic category 2001 census | Asian |
| 1751671000006110 | NHS Sickle Cell and Thalassaemia Screening Programme Pakistan family origin | Asian |
| 1968501000006111 | Asian or Asian Scottish or Asian British: Bangladeshi, Bangladeshi Scottish or Bangladeshi British - Scotland ethnic category 2011 census | Asian |
| 141521000000110 | Punjabi - ethnic category 2001 census | Asian |
| 158361000000116 | Asian and Chinese - ethnic category 2001 census | Asian |
| 286020010 | Asian - ethnic group | Asian |
| 1968521000006118 | Asian or Asian Scottish or Asian British: any other Asian group - Scotland ethnic category 2011 census | Asian |
| 141401000000117 | Chinese - ethnic category 2001 census | Asian |
| 4740361000006111 | Other ethnic, Asian/White origin | Asian |
| 1968491000006115 | Asian or Asian Scottish or Asian British: Indian, Indian Scottish or Indian British - Scotland ethnic category 2011 census | Asian |
| 142891000000115 | Sikh - ethnic category 2001 census | Asian |
| 1968341000006116 | Asian or Asian British: any other Asian background - Northern Ireland ethnic category 2011 census | Asian |
| 142881000000117 | Buddhist - ethnic category 2001 census | Asian |
| 1968151000006117 | Asian or Asian British: Bangladeshi - England and Wales ethnic category 2011 census | Asian |
| 157271000000119 | Indian or British Indian - ethnic category 2001 census | Asian |
| 141511000000116 | Mixed Asian - ethnic category 2001 census | Asian |
| 285991010 | Other ethnic, Asian/White orig | Asian |
| 196721000006111 | RACE: Pakistani | Asian |
| 286018012 | South East Asian | Asian |
| 1968321000006111 | Asian or Asian British: Bangladeshi - Northern Ireland ethnic category 2011 census | Asian |
| 4917941000006114 | Bangladesh | Asian |
| 250224013 | Asian origin | Asian |
| 1751681000006113 | NHS Sickle Cell and Thalassaemia Screening Programme Bangladesh family origin | Asian |
| 1751701000006111 | NHS Sickle Cell and Thalassaemia Screening Programme Chinese family origin | Asian |
| 1751661000006115 | NHS Sickle Cell and Thalassaemia Screening Programme India or African-Indian family origin | Asian |
| 196651000006112 | RACE: Chinese | Asian |
| 459784018 | Other Asian ethnic group | Asian |
| 1968331000006114 | Asian or Asian British: Chinese - Northern Ireland ethnic category 2011 census | Asian |
| 405069018 | E Afric Asian/Indo-Carib (NMO) | Asian |
| 141381000000117 | Other Asian background - ethnic category 2001 census | Asian |
| 1968141000006119 | Asian or Asian British: Pakistani - England and Wales ethnic category 2011 census | Asian |
| 937651000006117 | Other Asian or Asian unspecified - ethnic category 2001 census | Asian |
| 1968311000006115 | Asian or Asian British: Pakistani - Northern Ireland ethnic category 2011 census | Asian |
| 157351000000115 | Hindu - ethnic category 2001 census | Asian |
| 141541000000115 | East African Asian - ethnic category 2001 census | Asian |
| 56590016 | Chinese | Asian |
| 196611000006111 | Afro-Caucasian | Asian |
| 157301000000116 | Sinhalese - ethnic category 2001 census | Asian |
| 412016016 | O/E - Asian origin | Asian |
| 1751651000006117 | NHS Sickle Cell and Thalassaemia Screening Programme family origin South Asia (Asian) | Asian |
| 141621000000111 | Vietnamese - ethnic category 2001 census | Asian |
| 1968301000006118 | Asian or Asian British: Indian - Northern Ireland ethnic category 2011 census | Asian |
| 250228011 | Indian origin | Asian |
| 141631000000113 | Japanese - ethnic category 2001 census | Asian |
| 937541000006115 | Bangladeshi or British Bangladeshi - ethnic category 2001 census | Asian |
| 141551000000117 | Tamil - ethnic category 2001 census | Asian |
| 1968161000006115 | Asian or Asian British: Chinese - England and Wales ethnic category 2011 census | Asian |
| 1968511000006114 | Asian or Asian Scottish or Asian British: Chinese - Scotland ethnic category 2011 census | Asian |
| 1564291000006119 | Bangladeshi | Asian |
| 937941000006111 | Multi-ethnic islands: Mauritian or Seychellois or Maldivian or St Helena - ethnic category 2001 census | Asian |
| 196631000006117 | RACE: Bangladeshi | Asian |
| 142811000000112 | North African - ethnic category 2001 census | Black |
| 1968191000006111 | Black or African or Caribbean or Black British: Caribbean - England and Wales ethnic category 2011 census | Black |
| 250223019 | African origin | Black |
| 285931013 | Black, other, non-mixed origin | Black |
| 30683015 | Black African | Black |
| 285951012 | Black - other, mixed | Black |
| 285930014 | Black Caribbean | Black |
| 1751621000006114 | NHS Sickle Cell and Thalassaemia Screening Programme family origin African or African-Caribbean (black) | Black |
| 285943014 | Black - other African country | Black |
| 285950013 | Black Black - other | Black |
| 459782019 | Other black ethnic group | Black |
| 411584018 | Indo-Caribbean (NMO) | Black |
| 141601000000119 | Nigerian - ethnic category 2001 census | Black |
| 158351000000119 | Other Black background - ethnic category 2001 census | Black |
| 1968551000006110 | Caribbean or Black: Caribbean, Caribbean Scottish or Caribbean British - Scotland ethnic category 2011 census | Black |
| 285932018 | Black British | Black |
| 1968361000006117 | Black or African or Caribbean or Black British: Caribbean - Northern Ireland ethnic category 2011 census | Black |
| 405064011 | Black Caribbean/W.I./Guyana | Black |
| 453110019 | Black Guyana | Black |
| 411574019 | Black Arab | Black |
| 1968371000006112 | Black or African or Caribbean or Black British: other Black or African or Caribbean background - Northern Ireland ethnic category 2011 census | Black |
| 141391000000115 | African - ethnic category 2001 census | Black |
| 411576017 | Black East African Asian | Black |
| 250231012 | West Indian origin | Black |
| 285949013 | Black - other Asian | Black |
| 1968561000006112 | Caribbean or Black: Black, Black Scottish or Black British - Scotland ethnic category 2011 census | Black |
| 141571000000114 | Caribbean Asian - ethnic category 2001 census | Black |
| 1968351000006119 | Black or African or Caribbean or Black British: African - Northern Ireland ethnic category 2011 census | Black |
| 411579012 | West Indian (NMO) | Black |
| 1564491000006115 | Central African | Black |
| 1968571000006117 | Caribbean or Black: any other Black or Caribbean group - Scotland ethnic category 2011 census | Black |
| 286017019 | Other Pacific ethnic group | Black |
| 1968201000006114 | Black or African or Caribbean or Black British: other Black or African or Caribbean background - England and Wales ethnic category 2011 census | Black |
| 459730016 | Black - ethnic group | Black |
| 514611000006111 | Black Caribbean | Black |
| 157311000000119 | Black British - ethnic category 2001 census | Black |
| 1751641000006119 | NHS Sickle Cell and Thalassaemia Screening Programme African family origin | Black |
| 459731017 | Black | Black |
| 1968531000006115 | African: African, African Scottish or African British - Scotland ethnic category 2011 census | Black |
| 158371000000111 | Mixed Black - ethnic category 2001 census | Black |
| 154401000000118 | Caribbean - ethnic category 2001 census | Black |
| 514651000006112 | Black East African Asian/Indo-Caribbean | Black |
| 1968181000006113 | Black or African or Caribbean or Black British: African - England and Wales ethnic category 2011 census | Black |
| 411577014 | Black Indo-Caribbean | Black |
| 141591000000113 | Somali - ethnic category 2001 census | Black |
| 285948017 | Black Indian sub-continent | Black |
| 411573013 | Black North African | Black |
| 405065012 | Black N African/Arab/Iranian | Black |
| 405067016 | Caribbean I./W.I./Guyana (NMO) | Black |
| 285952017 | Other Black - Black/White orig | Black |
| 411575018 | Black Iranian | Black |
| 1968541000006113 | African: any other African - Scotland ethnic category 2011 census | Black |
| 937731000006115 | Other Black or Black unspecified - ethnic category 2001 census | Black |
| 285953010 | Other Black - Black/Asian orig | Black |
| 453109012 | Black West Indian | Black |
| 250243013 | Race: West indian | Black |
| 196601000006113 | Afro-Caribbean | Black |
| 141331000000116 | White and Black African - ethnic category 2001 census | Mixed |
| 141321000000118 | White and Black Caribbean - ethnic category 2001 census | Mixed |
| 285990011 | Other ethnic, Black/White orig | Mixed |
| 1968271000006115 | Mixed multiple ethnic groups: White and Black African - Northern Ireland ethnic category 2011 census | Mixed |
| 1968281000006117 | Mixed multiple ethnic groups: White and Asian - Northern Ireland ethnic category 2011 census | Mixed |
| 460153018 | Black Caribbean and White | Mixed |
| 141471000000113 | Black and Asian - ethnic category 2001 census | Mixed |
| 157291000000115 | Black and White - ethnic category 2001 census | Mixed |
| 1968291000006119 | Mixed multiple ethnic groups: any other Mixed or multiple ethnic background - Northern Ireland ethnic category 2011 census | Mixed |
| 460154012 | Black African and White | Mixed |
| 141341000000113 | White and Asian - ethnic category 2001 census | Mixed |
| 1968101000006116 | Mixed multiple ethnic groups: White and Black African - England and Wales ethnic category 2011 census | Mixed |
| 141481000000110 | Black and Chinese - ethnic category 2001 census | Mixed |
| 1968121000006114 | Mixed multiple ethnic groups: any other Mixed or multiple ethnic background - England and Wales ethnic category 2011 census | Mixed |
| 141491000000112 | Chinese and White - ethnic category 2001 census | Mixed |
| 141351000000111 | Other Mixed background - ethnic category 2001 census | Mixed |
| 285989019 | Other ethnic, mixed origin | Mixed |
| 1968111000006118 | Mixed multiple ethnic groups: White and Asian - England and Wales ethnic category 2011 census | Mixed |
| 1968261000006110 | Mixed multiple ethnic groups: White and Black Caribbean - Northern Ireland ethnic category 2011 census | Mixed |
| 1968091000006110 | Mixed multiple ethnic groups: White and Black Caribbean - England and Wales ethnic category 2011 census | Mixed |
| 459729014 | Mixed ethnic census group | Mixed |
| 937511000006119 | Other Mixed or Mixed unspecified - ethnic category 2001 census | Mixed |
| 4740401000006118 | Other ethnic, other mixed origin | Mixed |
| 1968471000006116 | Mixed or multiple ethnic groups: any Mixed or multiple ethnic group - Scotland ethnic category 2011 census | Mixed |
| 285993013 | Other ethnic, other mixed orig | Mixed |
| 4740341000006112 | Other ethnic, Black/White origin | Mixed |
| 138271000000119 | South and Central American - ethnic category 2001 census | Other |
| 138261000000114 | Iranian - ethnic category 2001 census | Other |
| 459785017 | Ethnic group | Other |
| 2484511000000112 | Ethnic category - 2011 census England and Wales | Other |
| 142901000000119 | Any other group - ethnic category 2001 census | Other |
| 649261000006110 | Ethnic group finding | Other |
| 4740241000006117 | British ethnic minority specified (NMO) | Other |
| 142851000000111 | Moroccan - ethnic category 2001 census | Other |
| 285988010 | Other ethnic NEC (NMO) | Other |
| 405068014 | N African Arab/Iranian (NMO) | Other |
| 4740261000006118 | British ethnic minority unspecified (NMO) | Other |
| 253628018 | O/E - ethnic origin | Other |
| 250229015 | Middle Eastern origin | Other |
| 4740421000006111 | Ethnicity / related nationality data | Other |
| 285960016 | Brit. ethnic minor. unsp (NMO) | Other |
| 141291000000111 | Ethnic category - 2001 census | Other |
| 411581014 | North African Arab (NMO) | Other |
| 141411000000115 | Other - ethnic category 2001 census | Other |
| 142841000000113 | Kurdish - ethnic category 2001 census | Other |
| 6270381000006117 | Ethnic groups (1991 census) (UK) | Other |
| 141421000000114 | Ethnic category not stated - 2001 census | Other |
| 1968211000006112 | Other ethnic group: Arab - England and Wales ethnic category 2011 census | Other |
| 6260901000006118 | Ethnic group | Other |
| 2484591000000115 | Ethnic category - 2011 census Scotland | Other |
| 253627011 | O/E - ethnic group | Other |
| 286005016 | Ethnicity and other related nationality data | Other |
| 8224821000006111 | Ethnicity | Other |
| 1968221000006116 | Other ethnic group: any other ethnic group - England and Wales ethnic category 2011 census | Other |
| 1968581000006119 | Other ethnic group: Arab, Arab Scottish or Arab British - Scotland ethnic category 2011 census | Other |
| 6270401000006117 | Ethnicity / related nationality data - finding | Other |
| 142861000000114 | Latin American - ethnic category 2001 census | Other |
| 285959014 | Brit. ethnic minor. spec.(NMO) | Other |
| 285958018 | Other ethnic non-mixed (NMO) | Other |
| 6270391000006119 | Ethnic groups (1991 census) (United Kingdom) | Other |
| 6591901000006115 | Ethnicity | Other |
| 1968381000006110 | Other ethnic group: Arab - Northern Ireland ethnic category 2011 census | Other |
| 1968591000006116 | Other ethnic group: any other ethnic group - Scotland ethnic category 2011 census | Other |
| 6597601000006118 | Ethnic background | Other |
| 2484551000000111 | Ethnic category - 2011 census Northern Ireland | Other |
| 138281000000117 | Muslim - ethnic category 2001 census | Other |
| 286003011 | Ethnic groups (census) NOS | Other |
| 2484471000000115 | Ethnic category - 2011 census | Other |
| 138251000000111 | Arab - ethnic category 2001 census | Other |
| 253635014 | O/E - ethnic group NOS | Other |
| 1968391000006113 | Other ethnic group: any other ethnic group - Northern Ireland ethnic category 2011 census | Other |
| 937871000006114 | Middle Eastern (excluding Israeli, Iranian and Arab) - ethnic category 2001 census | Other |
| 2484671000000118 | White: Irish - England and Wales ethnic category 2011 census | White |
| 2487361000000112 | White: Irish - Scotland ethnic category 2011 census | White |
| 937371000006116 | Other republics which made up the former Yugoslavia - ethnic category 2001 census | White |
| 142701000000116 | Greek - ethnic category 2001 census | White |
| 158481000000115 | Italian - ethnic category 2001 census | White |
| 141311000000112 | Other White background - ethnic category 2001 census | White |
| 286021014 | Other New Zealand ethnic group | White |
| 138201000000110 | Polish - ethnic category 2001 census | White |
| 142721000000113 | Turkish Cypriot - ethnic category 2001 census | White |
| 2486161000000112 | White - Northern Ireland ethnic category 2011 census | White |
| 1564391000006113 | British | White |
| 286009010 | Other European in New Zealand | White |
| 937301000006110 | Baltic States (Estonian or Latvian or Lithuanian) - ethnic category 2001 census | White |
| 141431000000111 | Scottish - ethnic category 2001 census | White |
| 1063981000000117 | White British - ethnic category 2001 census | White |
| 142781000000114 | Mixed Irish and other White - ethnic category 2001 census | White |
| 142831000000116 | Israeli - ethnic category 2001 census | White |
| 142691000000116 | Ulster Scots - ethnic category 2001 census | White |
| 1968051000006116 | White: English or Welsh or Scottish or Northern Irish or British - England and Wales ethnic category 2011 census | White |
| 459726019 | White British | White |
| 285925010 | White | White |
| 937311000006113 | Commonwealth of (Russian) Independent States - ethnic category 2001 census | White |
| 142761000000117 | Croatian - ethnic category 2001 census | White |
| 459728018 | White - ethnic group | White |
| 157991000000110 | Serbian - ethnic category 2001 census | White |
| 937411000006115 | Other White or White unspecified - ethnic category 2001 census | White |
| 1968081000006112 | White: any other White background - England and Wales ethnic category 2011 census | White |
| 285987017 | Other European (NMO) | White |
| 411595012 | Greek Cypriot (NMO) | White |
| 138191000000113 | Gypsy/Romany - ethnic category 2001 census | White |
| 405070017 | Greek/Greek Cypriot (NMO) | White |
| 4740381000006118 | Other ethnic, mixed white origin | White |
| 156921000000110 | Turkish - ethnic category 2001 census | White |
| 196641000006110 | Caucasian race | White |
| 937391000006115 | Other White European or European unspecified or Mixed European - ethnic category 2001 census | White |
| 286022019 | New Zealand ethnic group NOS | White |
| 2645811000000115 | Roma ethnic group | White |
| 1780407014 | White Scottish | White |
| 1968251000006113 | Irish Traveller - Northern Ireland ethnic category 2011 census | White |
| 138231000000116 | Albanian - ethnic category 2001 census | White |
| 286007012 | New Zealand European | White |
| 142751000000115 | Bosnian - ethnic category 2001 census | White |
| 286006015 | New Zealand ethnic groups | White |
| 2487281000000112 | White: Scottish - Scotland ethnic category 2011 census | White |
| 138171000000114 | Irish Traveller - ethnic category 2001 census | White |
| 142711000000119 | Greek Cypriot - ethnic category 2001 census | White |
| 1968441000006112 | White: Gypsy or Irish Traveller - Scotland ethnic category 2011 census | White |
| 141441000000119 | Welsh - ethnic category 2001 census | White |
| 1751821000006113 | NHS Sickle Cell and Thalassaemia Screening Programme family origin Northern European (white) | White |
| 141301000000110 | Irish - ethnic category 2001 census | White |
| 157281000000117 | English - ethnic category 2001 census | White |
| 141661000000115 | Cypriot (part not stated) - ethnic category 2001 census | White |
| 142741000000118 | Kosovan - ethnic category 2001 census | White |
| 141451000000116 | Northern Irish - ethnic category 2001 census | White |
| 285992015 | Other ethnic, mixed white orig | White |
| 1968071000006114 | White: Gypsy or Irish Traveller - England and Wales ethnic category 2011 census | White |
| 158341000000117 | British or mixed British - ethnic category 2001 census | White |
| 1780408016 | Other white British ethnic group | White |
| 2537217015 | Race: White | White |
| 2487481000000113 | White: Polish - Scotland ethnic category 2011 census | White |
| 405071018 | Turkish/Turkish Cypriot (NMO) | White |
| 411597016 | Turkish Cypriot (NMO) | White |
| 142791000000111 | Other mixed White - ethnic category 2001 census | White |
| 6846371000006111 | Caucasian | White |
| 2487321000000116 | White: other British - Scotland ethnic category 2011 census | White |
| 1968461000006111 | White: any other White ethnic group - Scotland ethnic category 2011 census | White |
| 138181000000111 | Traveller - ethnic category 2001 census | White |
| 141461000000118 | Cornish - ethnic category 2001 census | White |
| 1064041000000111 | White Irish - ethnic category 2001 census | White |
| 138241000000113 | Jewish - ethnic category 2001 census | White |
| 459727011 | White Irish | White |

Where a patient had inconsistent records for ethnicity, the most frequently occurring category for that patient was used. Ethnicity was recorded as ‘unknown’ if no category was most frequent or if no information was recorded.

## Supplementary Table 3a. Smoking status medical codes – CPRD GOLD

| **Medical code** | **Read term** | **Smoking status** |
| --- | --- | --- |
| 32973 | Chews tobacco | currently smoke |
| 12963 | Cigar consumption | currently smoke |
| 12943 | Cigar smoker | currently smoke |
| 12965 | Cigarette consumption | currently smoke |
| 46300 | Cigarette pack-years | currently smoke |
| 93 | Cigarette smoker | currently smoke |
| 10558 | Current smoker | currently smoke |
| 101338 | Failed attempt to stop smoking | currently smoke |
| 3568 | Heavy smoker - 20-39 cigs/day | currently smoke |
| 12964 | Keeps trying to stop smoking | currently smoke |
| 12944 | Light smoker - 1-9 cigs/day | currently smoke |
| 62686 | Minutes from waking to first tobacco consumption | currently smoke |
| 1878 | Moderate smoker - 10-19 cigs/d | currently smoke |
| 30762 | Not interested in stopping smoking | currently smoke |
| 12941 | Occasional smoker | currently smoke |
| 12947 | Pipe smoker | currently smoke |
| 12967 | Pipe tobacco consumption | currently smoke |
| 31114 | Ready to stop smoking | currently smoke |
| 46321 | Reason for restarting smoking | currently smoke |
| 12945 | Rolls own cigarettes | currently smoke |
| 1823 | Smoker | currently smoke |
| 12942 | Smoker - amount smoked | currently smoke |
| 12966 | Smoking reduced | currently smoke |
| 12951 | Smoking restarted | currently smoke |
| 41979 | Smoking restarted | currently smoke |
| 12952 | Smoking started | currently smoke |
| 30423 | Thinking about stopping smoking | currently smoke |
| 12960 | Tobacco consumption NOS | currently smoke |
| 12958 | Trivial smoker - < 1 cig/day | currently smoke |
| 12240 | Trying to give up smoking | currently smoke |
| 1822 | Very heavy smoker - 40+cigs/d | currently smoke |
| 105501 | Waterpipe tobacco consumption | currently smoke |
| 12878 | Date ceased smoking | formerly smoked |
| 19488 | Ex cigar smoker | formerly smoked |
| 26470 | Ex pipe smoker | formerly smoked |
| 100495 | Ex roll-up cigarette smoker | formerly smoked |
| 90 | Ex smoker | formerly smoked |
| 97210 | Ex-cigarette smoker | formerly smoked |
| 12956 | Ex-heavy smoker (20-39/day) | formerly smoked |
| 12957 | Ex-light smoker (1-9/day) | formerly smoked |
| 12955 | Ex-moderate smoker (10-19/day) | formerly smoked |
| 12946 | Ex-smoker - amount unknown | formerly smoked |
| 106891 | Ex-tobacco chewer | formerly smoked |
| 12961 | Ex-trivial smoker (<1/day) | formerly smoked |
| 12959 | Ex-very heavy smoker (40+/day) | formerly smoked |
| 99838 | Recently stopped smoking | formerly smoked |
| 776 | Stopped smoking | formerly smoked |
| 60 | Current non-smoker | never smoked |
| 33 | Never smoked tobacco | never smoked |
| 11788 | Non-smoker | never smoked |

## Supplementary Table 3b. Smoking status medical codes – CPRD Aurum

| **Medical code** | **Term** | **Smoking status** |
| --- | --- | --- |
| 482771000000118 | Smoking cessation drug therapy | currently smoke |
| 492511000000117 | Smoking cessation therapy | currently smoke |
| 3959111000006111 | Tobacco dependence syndrome | currently smoke |
| 250372012 | Trying to give up smoking | currently smoke |
| 102951000006115 | Tobacco dependence | currently smoke |
| 3419101000006116 | Moderate smoker (20 or less per day) | currently smoke |
| 5003151000006116 | Light cigarette smoker | currently smoke |
| 854961000006110 | Grade B light smoker (1-10/day) | currently smoke |
| 4980831000006112 | Finding relating to tobacco chewing | currently smoke |
| 854981000006117 | Grade C moderate smoker (11-20/day) | currently smoke |
| 504769011 | Chews tobacco | currently smoke |
| 604961000006114 | Current Smoker NOS | currently smoke |
| 7832511000006111 | Cigar | currently smoke |
| 108938018 | Cigarette smoker | currently smoke |
| 4074561000006112 | Tobacco smoke | currently smoke |
| 5003161000006119 | Moderate cigarette smoker | currently smoke |
| 5003171000006114 | Heavy cigarette smoker | currently smoke |
| 295256013 | Tobacco dependence, unspecified | currently smoke |
| 7832501000006113 | Cigarette | currently smoke |
| 88471000006112 | Trivial cigarette smoker (less than one cigarette/day) | currently smoke |
| 2669652019 | Smoking started | currently smoke |
| 5003141000006118 | Trivial cigarette smoker | currently smoke |
| 3430571000006116 | Tobacco | currently smoke |
| 5495901000006112 | Amount and type of tobacco smoked | currently smoke |
| 1538681000006118 | Smoke | currently smoke |
| 4948531000006116 | Smokes in bed | currently smoke |
| 250375014 | Rolls own cigarettes | currently smoke |
| 344793011 | Cigarette consumption | currently smoke |
| 13619901000006116 | Number of calculated smoking pack years | currently smoke |
| 852981000006111 | Rolls own cigarettes | currently smoke |
| 298701000000114 | History of tobacco use | currently smoke |
| 2670126018 | Smoking restarted | currently smoke |
| 8063181000006116 | Wants to stop smoking | currently smoke |
| 136515019 | Pipe smoker | currently smoke |
| 137791000006118 | Smoking restarted | currently smoke |
| 342574011 | Total time smoked | currently smoke |
| 854021000006115 | Cigarette smoker | currently smoke |
| 3142921000006110 | Tobacco | currently smoke |
| 3874641000006110 | Pipe smoking tobacco | currently smoke |
| 503483019 | Current smoker | currently smoke |
| 295258014 | Tobacco dependence, episodic | currently smoke |
| 128130017 | Smoker | currently smoke |
| 1484936019 | Smoking status at 52 weeks | currently smoke |
| 102921000006112 | Tobacco smoking consumption | currently smoke |
| 4980581000006110 | Age at starting smoking | currently smoke |
| 1780396011 | Cigarette pack-years | currently smoke |
| 6282331000006114 | Tobacco smoking behaviour - finding | currently smoke |
| 700121000006118 | Moderate cigarette smoker (10-19 cigs/day) | currently smoke |
| 1714541000006110 | Current smoker annual review - enhanced services admin | currently smoke |
| 961581000006114 | Smokes/uses tobacco products | currently smoke |
| 2170961000000116 | Waterpipe tobacco consumption | currently smoke |
| 4980741000006114 | Moist tobacco consumption | currently smoke |
| 6282371000006112 | Tobacco smoking consumption - finding | currently smoke |
| 4980781000006115 | User of moist powdered tobacco | currently smoke |
| 460828018 | Tobacco user | currently smoke |
| 295260011 | Tobacco dependence NOS | currently smoke |
| 397733018 | Occasional smoker | currently smoke |
| 295259018 | Tobacco dependence in remission | currently smoke |
| 4074571000006117 | Cigarette smoke | currently smoke |
| 3422221000006116 | Heavy smoker (over 20 per day) | currently smoke |
| 1484935015 | Smoking status between 4 and 52 weeks | currently smoke |
| 342445017 | Smokes drugs through a pipe | currently smoke |
| 2474719011 | Minutes from waking to first tobacco consumption | currently smoke |
| 3544141000006118 | Smoke | currently smoke |
| 819331000006110 | Heavy cigarette smoker (20-39 cigs/day) | currently smoke |
| 344794017 | Cigar consumption | currently smoke |
| 8153371000006117 | Occasional tobacco smoker | currently smoke |
| 1484934016 | Smoking status at 4 weeks | currently smoke |
| 99639019 | Cigar smoker | currently smoke |
| 344795016 | Pipe tobacco consumption | currently smoke |
| 743331000006116 | Light cigarette smoker (1-9 cigs/day) | currently smoke |
| 855001000006114 | Grade D heavy smoker (>20 Day) | currently smoke |
| 854071000006119 | Current smoker | currently smoke |
| 7375991000006118 | Smokes tobacco daily | currently smoke |
| 5003191000006110 | Chain smoker | currently smoke |
| 295257016 | Tobacco dependence, continuous | currently smoke |
| 904041000006113 | Waking time to first cigarette | currently smoke |
| 5003181000006112 | Very heavy cigarette smoker | currently smoke |
| 137771000006119 | Smoking Age Started | currently smoke |
| 5495951000006111 | Occasional cigarette smoker (less than one cigarette/day) | currently smoke |
| 250387019 | Tobacco consumption NOS | currently smoke |
| 1809121000006113 | Waterpipe tobacco consumption | currently smoke |
| 137711000006111 | Smoker (Read codes) | currently smoke |
| 1152111000000118 | Current smoker annual review | currently smoke |
| 11904991000006116 | Occasional cigarette smoker | currently smoke |
| 137721000006115 | Smoker - amount smoked | currently smoke |
| 6282351000006119 | Smoking | currently smoke |
| 67621000006112 | Very heavy cigarette smoker (40+ cigs/day) | currently smoke |
| 1819411000006114 | Smoking increased | currently smoke |
| 216212011 | Smoking reduced | currently smoke |
| 649821000006115 | Ex-cigar smoker | formerly smoked |
| 854051000006112 | Ex-pipe smoker | formerly smoked |
| 5496021000006114 | Tobacco smoking consumption unknown | formerly smoked |
| 418914010 | Ex-cigarette smoker | formerly smoked |
| 250363016 | Ex-trivial cigarette smoker (<1/day) | formerly smoked |
| 7368971000006117 | Stopped smoking before pregnancy | formerly smoked |
| 4980561000006117 | Time since stopped smoking | formerly smoked |
| 2636041000006110 | Cessation of smoking | formerly smoked |
| 2735201000000112 | Ex-very heavy smoker (40+/day) | formerly smoked |
| 649841000006110 | Ex-smoker | formerly smoked |
| 1817431000006112 | Tobacco use and exposure | formerly smoked |
| 903041000006110 | EX-Smoker NOS | formerly smoked |
| 7368961000006112 | Stopped smoking during pregnancy | formerly smoked |
| 250364010 | Ex-light cigarette smoker (1-9/day) | formerly smoked |
| 854151000006111 | Date stopped smoking | formerly smoked |
| 1123951000000110 | Ex-smoker annual review - enhanced services administration | formerly smoked |
| 649861000006114 | Ex-Cigarette Smoker | formerly smoked |
| 649851000006112 | Ex- Rolled Tobacco Smoker | formerly smoked |
| 137761000006114 | Smoking Age Ceased | formerly smoked |
| 250366012 | Ex-heavy cigarette smoker (20-39/day) | formerly smoked |
| 5495941000006114 | Occasional smoker | formerly smoked |
| 250373019 | Stopped smoking | formerly smoked |
| 1154471000000114 | Ex-smoker annual review | formerly smoked |
| 7368651000006119 | Smoked before confirmation of pregnancy | formerly smoked |
| 250371017 | Ex-smoker - amount unknown | formerly smoked |
| 250367015 | Ex-very heavy cigarette smoker (40+/day) | formerly smoked |
| 2735421000000119 | Ex-trivial smoker (<1/day) | formerly smoked |
| 2735281000000119 | Ex-heavy smoker (20-39/day) | formerly smoked |
| 2735331000000112 | Ex-moderate smoker (10-19/day) | formerly smoked |
| 137811000006119 | Smoking Status | formerly smoked |
| 1059701000000119 | Ex roll-up cigarette smoker | formerly smoked |
| 854111000006110 | Past smoker | formerly smoked |
| 1809131000006111 | Total time smoked | formerly smoked |
| 2735381000000111 | Ex-light smoker (1-9/day) | formerly smoked |
| 250365011 | Ex-moderate cigarette smoker (10-19/day) | formerly smoked |
| 2735181000000113 | Ex-smoker amount unknown | formerly smoked |
| 1151791000000117 | Recently stopped smoking | formerly smoked |
| 5496031000006112 | Ex-cigarette smoker amount unknown | formerly smoked |
| 8017571000006117 | Ex-smoker for more than 1 year | formerly smoked |
| 342602019 | Ex-tobacco chewer | formerly smoked |
| 6217151000006116 | Intolerant ex-smoker | formerly smoked |
| 6217281000006116 | Aggressive ex-smoker | formerly smoked |
| 649831000006117 | Ex-pipe smoker | formerly smoked |
| 1488873010 | Smoking free weeks | formerly smoked |
| 3513199018 | Ex-smoker for less than 1 year | formerly smoked |
| 853001000006110 | Ex-smoker NOS | formerly smoked |
| 250374013 | Current non-smoker | never smoked |
| 4980871000006110 | Never chewed tobacco | never smoked |
| 903051000006112 | Tobacco Consumption Nil | never smoked |
| 1123751000000113 | Non-smoker annual review - enhanced services administration | never smoked |
| 6718071000006115 | Current non smoker but past smoking history unknown | never smoked |
| 7965041000006111 | Never smoked any substance | never smoked |
| 5495921000006119 | Never smoked | never smoked |
| 14866014 | Non-smoker | never smoked |
| 1009271000006118 | Non Smoker - Nos | never smoked |
| 4980861000006115 | Does not chew tobacco | never smoked |
| 1154431000000112 | Non-smoker annual review | never smoked |
| 854951000006113 | Grade A non-smoker | never smoked |
| 397732011 | Never smoked tobacco | never smoked |
| 7569061000006118 | Never used tobacco | never smoked |
| 4980751000006111 | Does not use moist powdered tobacco | never smoked |

## Supplementary Table 4. Frequency of vaping codes per unique patient

| **Number of vaping codes per unique patient** | **Freq (n)** |
| --- | --- |
| 1 | 107,901 |
| 2 | 25,480 |
| 3 | 9,195 |
| 4 | 3,877 |
| 5 | 1,804 |
| >5 | 1,857 |
| **TOTAL unique patients** | **150,114** |

## Supplementary Table 5a. Previous and subsequent smoking status of patients who received a current vaping code

| **Previous smoking status** | **Subsequent (>12 months) smoking status** | **Current vaping code frequency, n (%) 147,130 (100.0)** | Proportion where 100% is the previous smoking status, Currently smoke: 80,986, 100% Formerly smoked: 56,300, 100% Never smoked: 8,211, 100% Unknown: 1,633, 100% |
| --- | --- | --- | --- |
| Currently smoke | Currently smoke | 27,703 (18.8) | 34.2 |
| Currently smoke | Formerly smoked | 19,209 (13.1) | 23.7 |
| Currently smoke | Never smoked | 1,384 (0.9) | 1.7 |
| Currently smoke | Unknown | 32,690 (22.2) | 40.4 |
| Formerly smoked | Currently smoke | 6,705 (4.6) | 11.9 |
| Formerly smoked | Formerly smoked | 21,017 (14.3) | 37.3 |
| Formerly smoked | Never smoked | 1,532 (1.0) | 2.7 |
| Formerly smoked | Unknown | 27,046 (18.4) | 48.0 |
| Never smoked | Currently smoke | 628 (0.4) | 7.7 |
| Never smoked | Formerly smoked | 1,547 (1.1) | 18.8 |
| Never smoked | Never smoked | 693 (0.5) | 8.4 |
| Never smoked | Unknown | 5,343 (3.6) | 65.1 |
| Unknown | Currently smoke | 186 (0.1) | 11.4 |
| Unknown | Formerly smoked | 294 (0.2) | 18.0 |
| Unknown | Never smoked | 39 (0.0) | 2.4 |
| Unknown | Unknown | 1,114 (0.8) | 68.2 |

## Supplementary Table 5b. Previous and subsequent smoking status of patients who received a former vaping code

| **Previous smoking status** | **Subsequent (>12 months) smoking status** | **Former vaping code frequency, n (%) 5,147 (100.0)** | Proportion where 100% is the previous smoking status, Currently smoke: 3,127, 100% Formerly smoked: 1,779, 100% Never smoked: 204, 100% Unknown: 37, 100% |
| --- | --- | --- | --- |
| Currently smoke | Currently smoke | 1,357 (26.4) | 43.4 |
| Currently smoke | Formerly smoked | 606 (11.8) | 19.4 |
| Currently smoke | Never smoked | 56 (1.1) | 1.8 |
| Currently smoke | Unknown | 1,108 (21.5) | 35.4 |
| Formerly smoked | Currently smoke | 229 (4.5) | 12.9 |
| Formerly smoked | Formerly smoked | 708 (13.8) | 39.8 |
| Formerly smoked | Never smoked | 49 (1.0) | 2.8 |
| Formerly smoked | Unknown | 793 (15.4) | 44.6 |
| Never smoked | Currently smoke | 27 (0.5) | 13.2 |
| Never smoked | Formerly smoked | 39 (0.8) | 19.1 |
| Never smoked | Never smoked | 28 (0.5) | 13.7 |
| Never smoked | Unknown | 110 (2.1) | 53.9 |
| Unknown | Currently smoke | 8 (0.2) | 21.6 |
| Unknown | Formerly smoked | 12 (0.2) | 32.4 |
| Unknown | Never smoked | 2 (0.0) | 5.4 |
| Unknown | Unknown | 15 (0.3) | 40.5 |

## Supplementary Graph 1: Patient-level first-time incidence of current vaping and former medical codes, by region


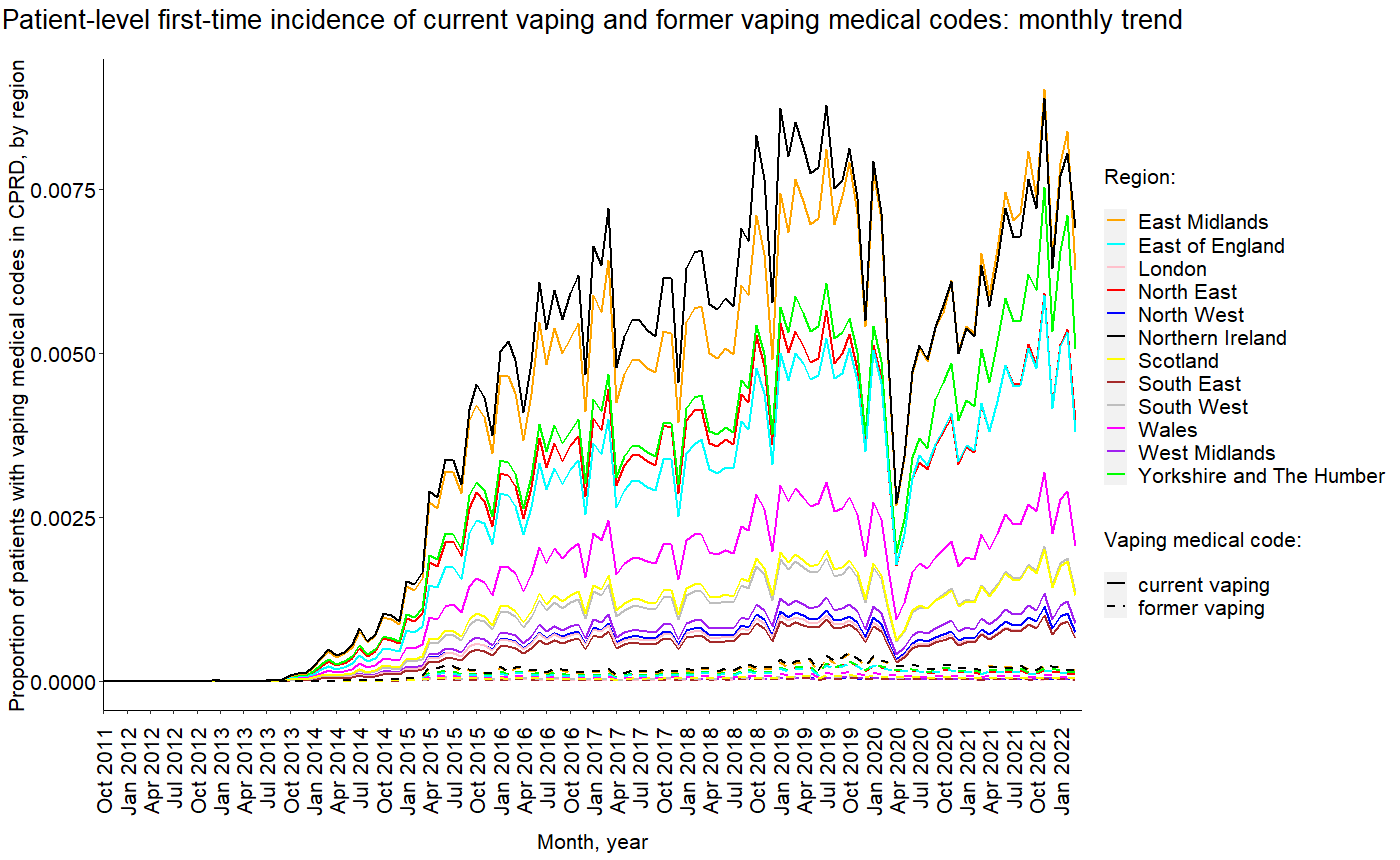


## Supplementary Graph 2: Transition between previous smoking status and subsequent (>12 months) smoking status of patients when they received their first former vaping code

The ‘nodes’ (vertical bars) are coloured to represent the smoking status record obtained in the consultation (red: currently smoke, green: formerly smoked, blue: never smoked, unknown: grey). The ‘connections’ (transitions from left to right) are coloured to represent the previous smoking status (red: currently smoke, green: formerly smoked, blue: never smoked, unknown: grey).

The + signs on the right side (subsequent smoking status) indicate the proportion breakdown of previous smoking status categories. For example: Those who ‘currently smoke’ before receiving the former vaping code, >12 months after they received the former vaping code: 43.4% of them were currently smoking, 19.4% of them had quit smoking, 1.8% received a ‘never smoked’ code, and 35.4% had no smoking status recorded. (43.4% + 19.4% + 1.8% + 35.4 = 100%)

The mean time difference between the previous smoking status record and the former vaping code record was 430.6 days (SD: 535.7, range: 1.0 to 9,276.0, median: 313.5). The mean time difference between the subsequent smoking status record and the former vaping code record was 1,101.3 days (SD: 557.4, range: 366.0 to 2,761.0, median: 970.0).


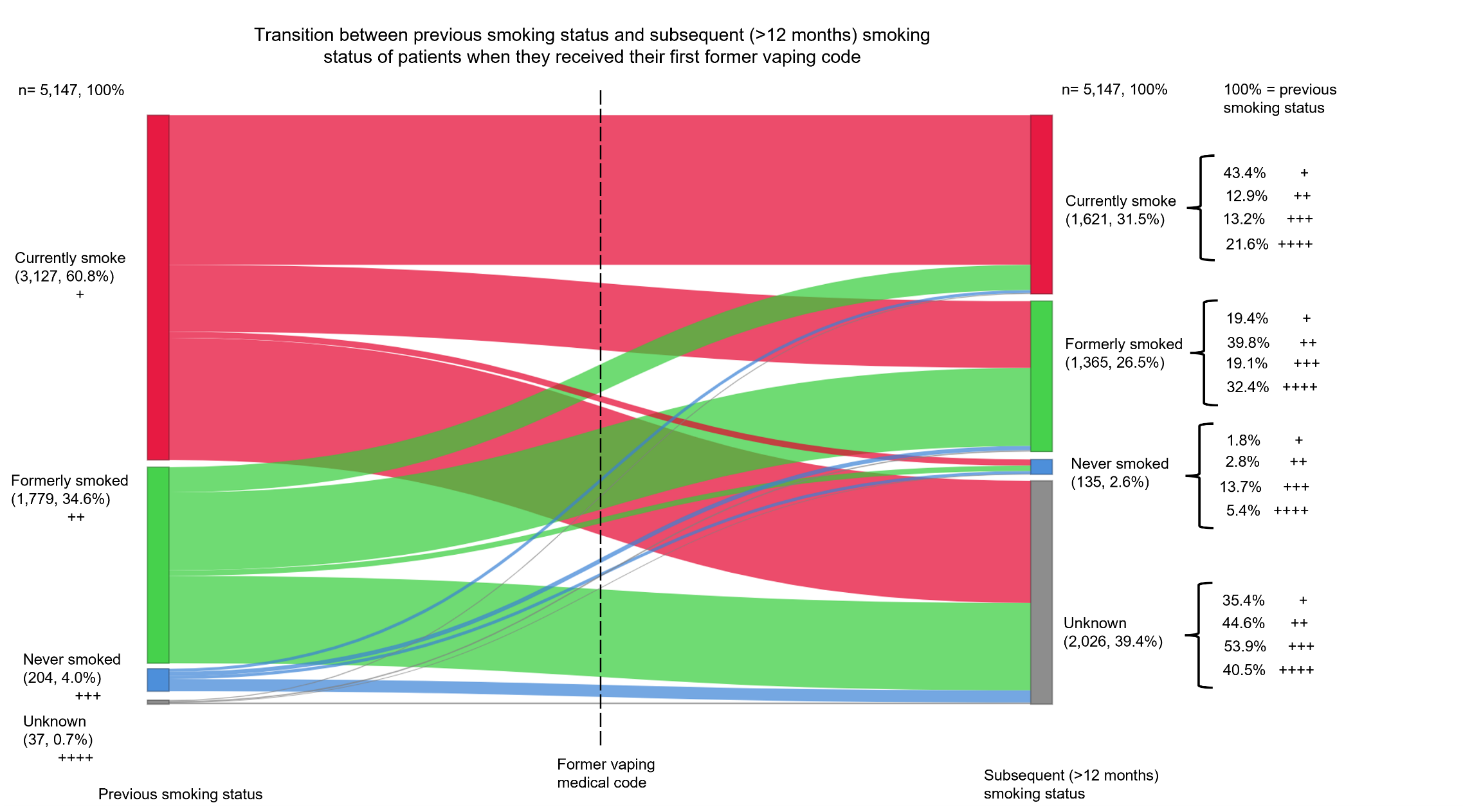


## Supplementary Graph 3: Transition between previous smoking status and subsequent (>12–≤24 months) smoking status of patients when they received their first current vaping code

The ‘nodes’ (vertical bars) are coloured to represent the smoking status record obtained in the consultation (red: currently smoke, green: formerly smoked, blue: never smoked, unknown: grey). The ‘connections’ (transitions from left to right) are coloured to represent the previous smoking status (red: currently smoke, green: formerly smoked, blue: never smoked, unknown: grey).

The + signs on the right side (subsequent smoking status) indicate the proportion breakdown of previous smoking status categories. For example: Those who ‘currently smoke’ before receiving the current vaping code, >12–≤24 months after they received the current vaping code: 30.2% of them were currently smoking, 16.9% of them had quit smoking, 0.9% received a ‘never smoked’ code, and 52.1% had no smoking status recorded. (30.2% + 16.9% + 0.9% + 52.1% = 100%)

The mean time difference between the previous smoking status record and the current vaping medical code record was 542.6 days (SD: 668.1 days, range: 1.0 to 14,729.0, median: 344.0). The mean time difference between the subsequent smoking status record and the current vaping medical code record was 564.1 days (SD: 111.8, range: 366.0 to 730.0, median: 574.0).


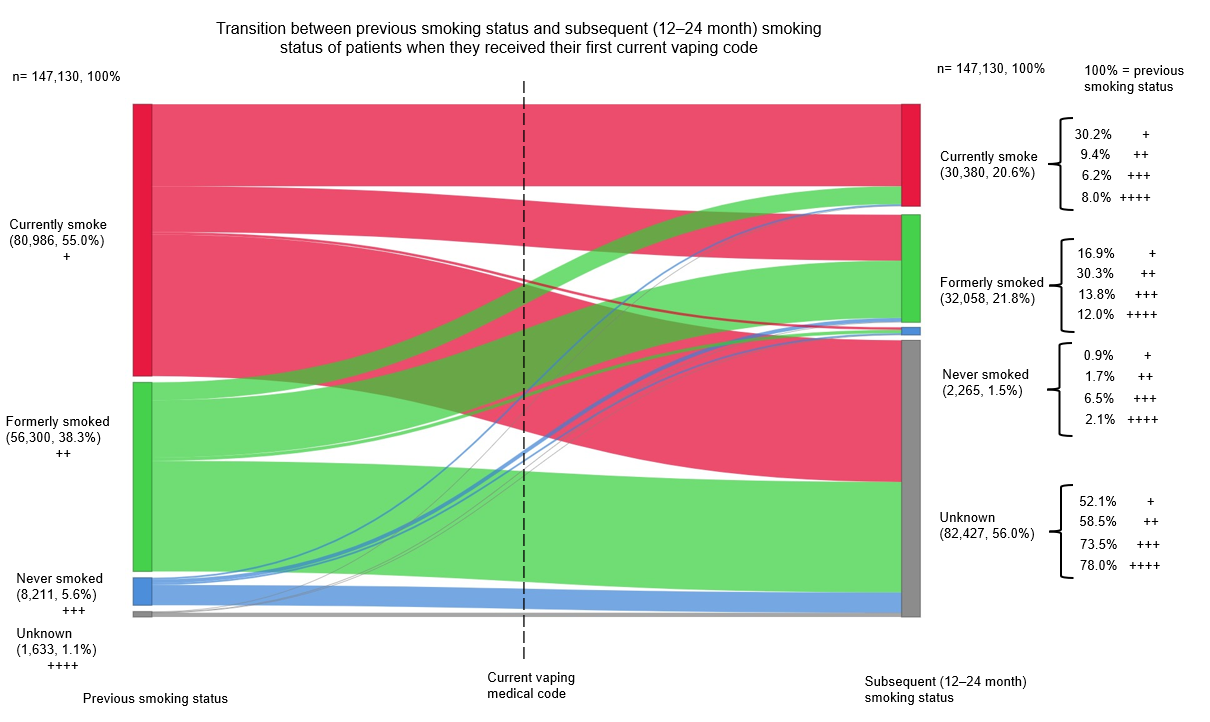

Supplement: Supplementary file 1 — Supplementary Material 1 [file 12889_2023_17200_MOESM1_ESM.docx]
